# Simvastatin in liver diseases: therapeutic value and research advances

**DOI:** 10.1007/s10238-026-02149-9

**Published:** 2026-04-19

**Authors:** Haodong Zhang, Lei Xu, Yong Cui, Wei Gou, Jinjin Li

**Affiliations:** 1 Department of Metabolic Hepatology, Qingdao Public Health Clinical Center, Qingdao, 266000 China; 2 Internal Medicine (Cadre Healthcare Department), Qingdao Public Health Clinical Center, Qingdao, 266000 China; 3 Unit 92886, Qingdao, 266000 China; 4 Hepatobiliary Oncology, Qingdao Public Health Clinical Center, No. 9 Fushun Road, Shibei District, 266000 Qingdao, Shandong China

## Introduction

### Disease burden of full-cycle chronic liver disease and unmet clinical needs

Chronic hepatitis, liver cirrhosis, and hepatocellular carcinoma (HCC) constitute the complete disease spectrum of the progressive progression of chronic liver disease, which is a major public health problem threatening the health of the global population. Among them, metabolic dysfunction-associated steatotic liver disease (MASLD) is currently the most common chronic liver disease worldwide, with a global adult prevalence of approximately 38% and a pooled prevalence of 29.6% in China. Its disease process can gradually progress from simple hepatocellular steatosis to metabolic dysfunction-associated steatohepatitis (MASH), liver fibrosis, and even liver cirrhosis, while increasing the risk of onset and death of type 2 diabetes mellitus (T2DM) and cardiovascular disease [1–3]. Liver cirrhosis, as the end-stage pathological outcome of various types of chronic liver injury, is characterized by the progressive accumulation of liver fibrosis, destruction of intrahepatic microcirculation structure, and persistent elevation of portal hypertension, and is the main cause of fatal complications such as ascites, esophagogastric variceal bleeding, and hepatorenal syndrome [4]. Liver cirrhosis can further progress to liver cancer, among which HCC accounts for more than 90% of primary liver cancers and ranks among the leading causes of cancer-related death worldwide [5].

There are still a large number of unmet clinical needs in the full-cycle diagnosis and treatment of chronic liver disease: there is no approved specific therapeutic drug worldwide for the MASLD stage, and existing management methods cannot effectively block the progression of the disease to liver fibrosis and liver cirrhosis [6, 7]; there is no approved disease-modifying therapeutic drug that can reverse liver fibrosis and improve long-term prognosis in the liver cirrhosis stage, traditional treatments for portal hypertension have limitations such as systemic hemodynamic effects, and end-stage patients can only rely on liver transplantation with extremely low accessibility [8, 9]; in the whole chain of HCC prevention and treatment, primary prevention methods for high-risk groups are limited, the 5-year recurrence rate after radical resection can reach 70% without approved adjuvant therapy regimens, the objective response rate of targeted and immunotherapy for advanced patients is only 15%~20%, and drug resistance and immunosuppressive microenvironment are still the core clinical pain points [5, 10, 11]. In this context, exploring the hepatoprotective effects of marketed drugs and expanding their clinical application value has become an important direction to fill the gap in the diagnosis and treatment of chronic liver disease [12].

### Pharmacological basis of simvastatin and research overview in the field of liver disease

Simvastatin is a classic competitive inhibitor of 3-hydroxy-3-methylglutaryl coenzyme A (HMG-CoA) reductase approved by the U.S. Food and Drug Administration (FDA). With decades of clinical application, its safety and tolerability have been fully verified. Its core approved indication is the regulation of dyslipidemia. It exerts its core lipid-lowering pharmacological effect by inhibiting HMG-CoA reductase, the rate-limiting enzyme of cholesterol synthesis, and blocking the biosynthesis of endogenous cholesterol in the liver [13].

In recent years, a large number of preclinical and clinical evidence has found that simvastatin has potential benefits of delaying the progression of liver fibrosis and reducing the risk of decompensation events and long-term adverse outcomes in patients with MASLD complicated with hyperlipidemia and T2DM, and in populations with compensated cirrhosis. However, the international multicenter phase 3 randomized controlled clinical trial (LIVERHOPE study, JAMA 2023) clearly confirmed that simvastatin combined with rifaximin failed to reduce the risk of acute-on-chronic liver failure (ACLF), liver transplantation/all-cause mortality, and the incidence of liver cirrhosis complications in the general population with decompensated cirrhosis, and did not confirm the benefit of disease-modifying therapy, which is the core evidence-based red line for clinical application in this population.

#### Special Statement

**Fig. 1:**
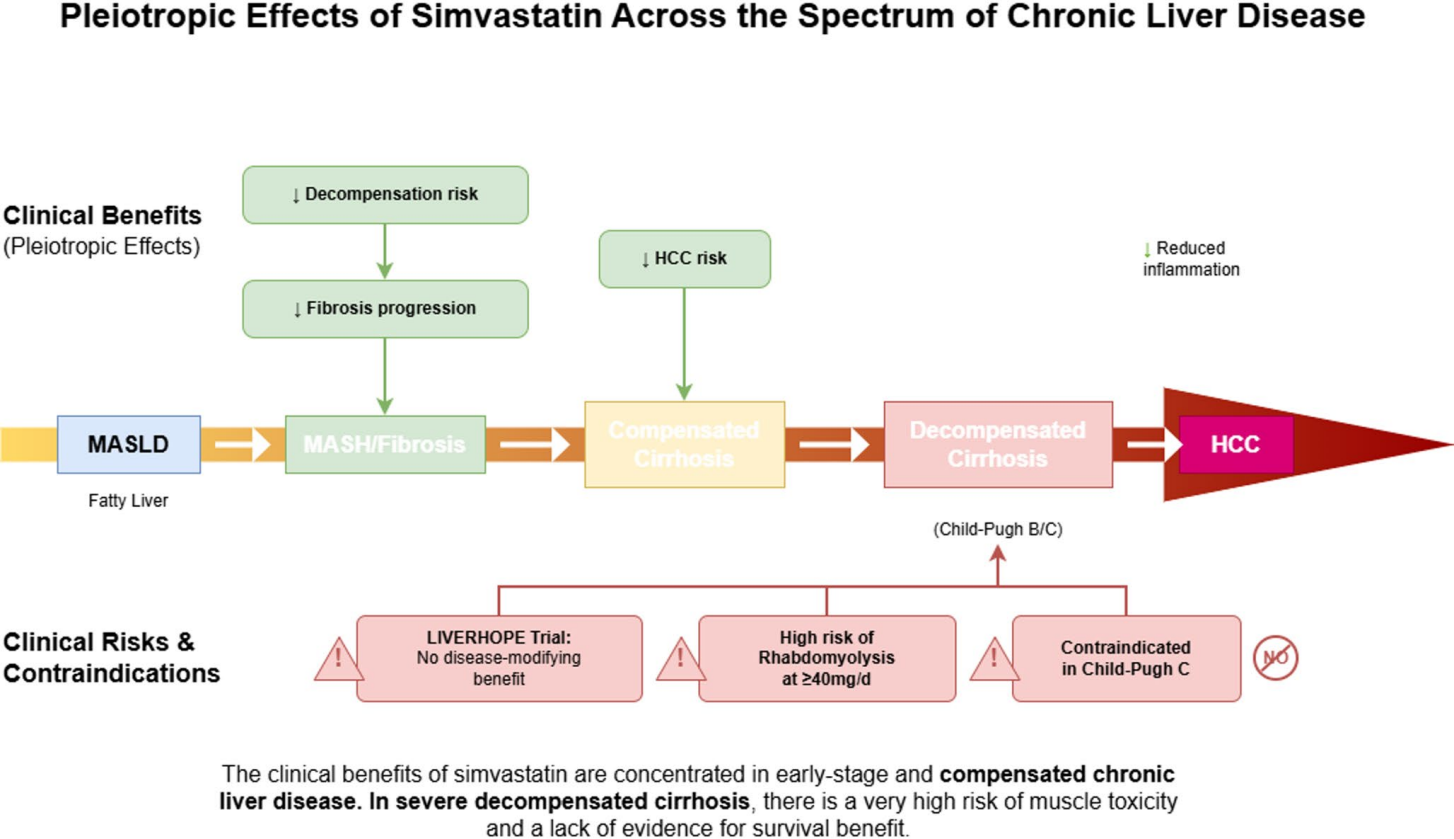
Pleiotropic Effects of Simvastatin Across the Spectrum of Chronic Liver Disease

The only globally approved legal indication of simvastatin is the regulation of dyslipidemia. The liver-related applications in chronic liver disease described in this article all belong to the category of off-label drug use. Clinical application must strictly abide by the evidence boundary and make prudent decisions based on individualized benefit-risk assessment (Table 1) Fig 1.

**Table 1:** Summary of core clinical evidence-based studies of simvastatin in chronic liver disease

Core Study/Source Literature	Disease Field	Study Design and Population Size	Intervention/Exposure Regimen	Core Conclusions and Clinical Guiding Implications
Chen et al. (2024)	MASLD / HCC Primary Prevention	Large-sample retrospective cohort < br> NHIRD Database < br > *N* = 275,790 (patients with T2DM)	Statin exposure < br> (simvastatin as one of the main drugs)	Positive benefit: Risk of decompensated liver cirrhosis (DLC) ↓32%, risk of HCC ↓40%.< br> Implication: Confirmed that the protective effect of statins is dose-dependent, and established the long-term clinical hard endpoint benefit in the population with T2DM complicated with MASLD.
Noureddin et al. (2021)	MASLD Fibrosis Delay	Single-center retrospective cohort < br> Cleveland Clinic, USA < br > *N* = 1,183 (confirmed by liver biopsy)	Exposure to simvastatin and other statins	Positive benefit: Risk of advanced liver fibrosis (stage 3–4) ↓39%.< br> Implication: Provided observational evidence of histological benefit based on the “gold standard of liver biopsy”.
Kamal et al. (2024)	Compensated Cirrhosis Prevention	Large-sample retrospective cohort < br > US Veterans Affairs (VA) Database < br > *N* = 335,991	Exposure to simvastatin and other statins < br> (annual medication days > 245 days)	Positive benefit: Risk of new-onset liver cirrhosis ↓26%.< br> Implication: Confirmed that the benefit is more significant in older patients, and the equivalent of 20 mg/d simvastatin can achieve significant protection.
Mahmud et al. (2023)	ACLF Primary Prevention	Large-sample retrospective cohort < br > US Veterans Affairs (VA) Database < br > *N* = 84,963 (new-onset cirrhosis)	Exposure to simvastatin and other statins < br> (more optimal benefit in the ≥ 20 mg/d group)	Positive benefit: Risk of high-grade ACLF ↓38%, and reduced short-term mortality.< br> Implication: Revealed the dose-effect relationship between cumulative exposure duration and decompensation prevention.
Abraldes et al. (2009)	Liver Cirrhosis and Portal Hypertension	Multicenter double-blind RCT < br > *N* = 59 (HVPG≥12mmHg)	Simvastatin (20 mg increased to 40 mg/d) vs. placebo, 1 month	Positive benefit: HVPG significantly decreased by 8.3%, and 32% of patients achieved hemodynamic response.< br> Implication: Established the short-term effectiveness of simvastatin in reducing portal pressure, without affecting systemic hemodynamics.
Rodrigues et al. (2023)	Liver Cirrhosis and Portal Hypertension	Double-blind RCT < br > *N* = 82 (patients with poor response to NSBBs)	Carvedilol + simvastatin vs. carvedilol + placebo	Positive benefit: The combined group had a greater reduction in HVPG, and the response rate increased from 15% to 37%.< br> Implication: Provided an efficacy-enhancing regimen for high-risk patients with poor response to traditional β-blockers.
BLEPS Study (2016)	Secondary Prevention in Decompensated Stage	Multicenter double-blind RCT < br > *N* = 158 (after variceal bleeding)	Standard treatment + simvastatin (40 mg/d) vs. placebo, up to 24 months	Survival benefit: Did not reduce the rebleeding rate, but the relative risk of all-cause mortality ↓61%.< br> Risk warning: The 40 mg/d dose caused rhabdomyolysis in patients with Child-Pugh grade C.
Kumar et al. (2024)	Secondary Prevention in Decompensated Stage	Open-label RCT < br > *N* = 268 (after variceal bleeding)	Standard treatment + simvastatin (20 mg/d) vs. standard treatment, 24 months	Survival benefit: 24-month all-cause mortality risk ↓52% (NNT = 8), and the risk of ascites decreased.< br> Implication: Confirmed that the low dose of 20 mg/d is safe and can improve long-term survival in patients with mild to moderate decompensation.
LIVERHOPE Study	Disease Modification in Decompensated Stage	International multicenter phase 3 RCT < br > *N* = 237 (Child-Pugh grade B/C)	Simvastatin (20 mg/d) + rifaximin vs. placebo, 12 months	Negative result: Did not reduce the risk of ACLF, and no improvement in liver cirrhosis complications and mortality.< br> Clinical red line: Does not support routine use for disease-modifying therapy in the general population with severe decompensated cirrhosis.

## Application of simvastatin in metabolic dysfunction-associated steatotic liver disease

### Anchoring of clinical pain points

MASLD can be the initial link of the full cycle of chronic liver disease, and the core disease progression process is simple steatosis→MASH→liver fibrosis→liver cirrhosis/HCC. At present, there is no approved specific therapeutic drug worldwide, and the core clinical need is to block disease progression and reduce the risk of end-stage liver disease events and cardiovascular events [3, 14]. The lipid-lowering pleiotropic effects of simvastatin can cover the three core pathological links of MASLD: lipid disorder, inflammation, and fibrosis. The following stratified presentation of its evidence-based evidence, mechanism of action, and clinical application boundaries.

### Clinical evidence-based evidence

This section is strictly sorted by the level of evidence from high to low. Each study simultaneously presents the core results and key limitations to clarify the evidence boundary.

#### Long-term clinical hard endpoint benefit: clinical evidence of reducing the occurrence of liver cirrhosis and HCC

The core evidence of simvastatin for MASLD-related long-term clinical hard endpoints comes from large-sample real-world cohort studies, and there is a lack of prospective RCT data on clinical hard endpoints.

(1) Decompensated liver cirrhosis (DLC) endpoint: A retrospective cohort study based on the National Health Insurance Research Database (NHIRD) included patients with T2DM complicated with non-viral liver disease who were diagnosed between 2008 and 2020 and aged ≥ 40 years. After 1:1 propensity score matching (PSM), 275,790 subjects were included (137,895 statin users and 137,895 non-users), and were followed up until December 31, 2021. The results showed that simvastatin monotherapy significantly reduced the risk of MASLD-related decompensated liver cirrhosis by 32% in patients with T2DM (adjusted hazard ratio aHR = 0.68, 95%CI 0.61–0.77, *P* < 0.0001); the incidence of DLC in simvastatin users was 14.25 per 10,000 person-years, which was significantly lower than 21.94 per 10,000 person-years in non-statin users [15]. The study also clarified that the protective effect of simvastatin showed a significant dose-dependent manner, and only when the cumulative dose reached a certain threshold did it show a significant benefit, while low-dose medication had no significant protective effect. There was a U-shaped dose-effect relationship between daily medication intensity and DLC risk, and the optimal daily medication intensity was 0.88 defined daily doses (DDD).

(2) Hepatocellular carcinoma (HCC) endpoint: The same large-sample cohort study mentioned above showed that simvastatin significantly reduced the risk of HCC by 40% in patients with T2DM (adjusted hazard ratio aHR = 0.60, 95%CI 0.47–0.77, *P* < 0.0001), and it was one of the only 3 commonly used statins with a significant protective effect on HCC; its protective effect on HCC was also dose-dependent, only medium and high cumulative doses could significantly reduce the risk of HCC, and low doses had no significant benefit [15].

(Note: The above data on the protective effect on the incidence risk of DLC and HCC are based on large-sample retrospective cohorts. For the exact causal relationship and the extrapolation boundary in non-diabetic and non-Asian populations, see Sect.  6.1 of this article.)

#### Clinical evidence of liver fibrosis endpoint

(1) Fibrosis endpoint confirmed by liver biopsy: A single-center retrospective cohort study conducted at the Cleveland Clinic in the United States included 1,183 patients with T2DM complicated with MASLD confirmed by liver biopsy, with the core endpoint of advanced liver fibrosis (stage 3–4). After adjusting for confounding factors such as age, gender, race, and BMI, the results showed that simvastatin use was significantly associated with a reduced risk of advanced liver fibrosis (OR = 0.61, 95%CI 0.44–0.85, *P* = 0.003), which could reduce the risk of advanced fibrosis by 39% in patients with T2DM complicated with MASLD [16]. Advantages and limitations of the study: It adopted the gold standard endpoint of liver biopsy fibrosis, with a higher level of evidence than studies on non-invasive markers; however, it was a single-center retrospective design, with recall bias and unknown confounding factors, and could not confirm a causal relationship.

A European multicenter cohort study included 107 subjects who completed liver puncture and received statin therapy for ≥ 6 months, among which simvastatin had the highest use rate of 49%. After multivariate adjustment for confounding factors such as age, gender, BMI, abnormal glucose metabolism, core genetic risk variants of MASLD (PNPLA3/TM6SF2), and blood lipid levels, statin use dominated by simvastatin was significantly associated with a 41% reduction in the risk of clinically significant fibrosis (stage F2-F4) (OR = 0.59, 95% CI 0.34–0.98, *P* = 0.041); and the fibrosis protective effect showed a clear dose-dependent manner, with the risk of fibrosis progressively decreasing with the increase of statin treatment intensity (*P* = 0.028). A nested case-control analysis with 1:1 strict matching of demographic, metabolic, genetic and other confounding factors further verified that statin use reduced the risk of stage F2-F4 fibrosis by 58% (OR = 0.42, 95% CI 0.20–0.85, *P* = 0.017); in the sensitivity analysis matched within the recruitment center, the reduction in fibrosis risk reached 63% (OR = 0.37, 95% CI 0.16–0.87, *P* = 0.016), and a significant protective effect on stage F3-F4 advanced fibrosis was observed for the first time (OR = 0.42, 95% CI 0.17–0.99, *P* = 0.049) [17].

(Note: Although the above conclusions have the advantage of the gold standard of liver biopsy histology, they are limited by single-center or retrospective design and sample size. For the discussion of the exact causal relationship, see Sect.  6.1 of this article.)

(2) Evidence related to non-invasive fibrosis markers: In the existing included literature, there is a lack of large-sample clinical research data of simvastatin on non-invasive fibrosis markers (such as FIB-4, MASLD fibrosis score NFS, liver stiffness measurement) in patients with MASLD. Only small-sample clinical observations suggest that simvastatin can reduce the level of serum markers related to liver fibrosis, and there is no high-quality clinical evidence to support it.

#### Clinical evidence of metabolic and biochemical surrogate endpoint indicators

(1) Blood lipid and liver function biochemical indicators: A single-center clinical controlled study in China included 168 patients with MASLD complicated with T2DM, divided into metformin monotherapy group (75 cases) and metformin combined with simvastatin group (93 cases, simvastatin 20 mg/d), with an intervention cycle of 3 months. The results showed that combined simvastatin therapy significantly reduced the levels of serum total cholesterol (TC) and triglyceride (TG), and increased the level of high-density lipoprotein cholesterol (HDL-C) in patients (all *P* < 0.05); there was no significant difference in serum ALT and AST levels between the two groups after treatment [18]. A number of preclinical animal experiments have consistently confirmed that simvastatin can significantly reduce the serum ALT and AST levels of MASLD model animals and reduce hepatocyte injury [19, 20], but this effect has not been consistently verified in large-sample human clinical trials.

(2) Insulin resistance and glucose metabolism-related indicators: The above domestic clinical controlled study showed that after 3 months of combined simvastatin treatment, the homeostatic model assessment for insulin resistance (HOMA-IR) of patients was significantly lower than that of the metformin monotherapy group (*P* < 0.05), suggesting that simvastatin can further improve insulin resistance in patients with MASLD complicated with T2DM; while there was no significant difference in glycosylated hemoglobin (HbA1c) and fasting insulin (FINS) levels between the two groups [18].

In the MASH rat model induced by high-fat and high-sugar diet, simvastatin can completely reverse insulin resistance in model animals and restore fasting blood glucose and insulin levels to the normal range [21], but this effect has not been verified by large-sample human studies.

(3) Clinical evidence related to hepatic steatosis: In existing clinical studies, there is a lack of large-sample high-quality clinical data of simvastatin on the quantitative imaging assessment (such as MRI-PDFF) of hepatic steatosis and the improvement of histological steatosis score of the liver in human patients with MASLD. Only preclinical animal experiments have confirmed that simvastatin can significantly reduce hepatic steatosis [20, 22], and the human clinical evidence is insufficient.

(Note: The above conclusions on the improvement of blood lipids and insulin resistance are mainly based on small-sample, short-cycle clinical observations. Whether they can be transformed into long-term clinical hard endpoint benefits remains to be confirmed, see Sect.  6.1 of this article.)

### Core biological mechanisms corresponding to clinical benefits

#### Core mechanisms of Anti-fibrosis

(1) Repairing the function of liver sinusoidal endothelial cells (LSECs) and improving hepatic sinusoidal microcirculation disturbance [Evidence from preclinical animal experiments and in vitro cell experiments]: LSECs are the most abundant non-parenchymal cells in the liver. Differentiated LSECs in a healthy state can maintain the quiescence of hepatic stellate cells (HSCs); after liver injury, capillarization of LSECs (loss of fenestral structure, formation of basement membrane) occurs, which is an early initiating event of liver fibrosis, precedes the activation of HSCs, and is a key link driving the progression of fibrosis [23]. Simvastatin can up-regulate the expression of Rab7 protein, promote the fusion of autophagosome and lysosome, maintain autophagic flux, and then up-regulate the expression of KLF2, maintain the differentiated phenotype of LSEC, and reverse its capillarization changes by activating the KLF2-autophagy positive feedback regulatory loop [24]; at the same time, it can reduce the proportion of dedifferentiated CD32b- LSECs, inhibit the overexpression of vasoconstrictors such as ET-1, restore the endothelium-dependent vasodilation function of the liver, and improve hepatic sinusoidal microcirculation disturbance [21]; it can also up-regulate the expression and activity of endothelial nitric oxide synthase (eNOS), increase the bioavailability of NO, maintain hepatic sinusoidal vascular homeostasis, and reduce leukocyte adhesion and recruitment in the hepatic sinus [24, 25].

(2) Inhibiting the activation of HSCs and regulating the synthesis of extracellular matrix (ECM) [Evidence from preclinical animal experiments and in vitro cell experiments]: Hepatic stellate cells (HSCs) are the core effector cells in the occurrence and development of liver fibrosis. Their phenotypic transformation, metabolic reprogramming and intercellular interaction are the core links driving the progression of chronic liver injury to liver fibrosis and even cirrhosis [26]; the abnormal deposition of extracellular matrix (ECM) is the core pathological event of this process, which is not only the pathological product of fibrotic remodeling of liver tissue, but also further drives disease progression by remodeling the mechanical microenvironment of the liver [27].

Simvastatin can inhibit the activation of HSCs and regulate extracellular matrix through multi-channel synergistic effects. The core mechanisms include:

① Blocking the inducing factors of activation upstream: Reversing the dedifferentiated phenotype of LSECs, inhibiting the overexpression of endothelin-1 (ET-1), and eliminating the key driving factors of HSC activation; ② Core pathway inhibition: Blocking the RAF/MEK/ERK cascade reaction downstream of ET-1, and simultaneously inhibiting the RhoA/Rho kinase and Ras/ERK pathways, exerting the core anti-fibrosis effect; ③ Improving the pro-activation microenvironment: Restoring the activity of antioxidant enzymes, down-regulating the ALE-RAGE stress axis and iNOS expression, and alleviating the oxidative stress and inflammatory microenvironment that drive HSC activation; ④ Final biological effect: Through the above mechanisms, significantly down-regulate the expression of pro-fibrotic genes such as α-SMA and Col1a1 in HSCs, and restore the quiescent phenotype of HSCs with vitamin A storage capacity. ⑤ At the same time, directly down-regulate the gene transcription of type I collagen α1, the core effector molecule of fibrosis, reduce the synthesis and pathological deposition of extracellular matrix such as collagen, and finally reverse the pathological progression of liver fibrosis and maintain the homeostasis of liver extracellular matrix [21, 25, 28].

#### Core mechanisms of regulating lipid metabolism, Anti-inflammation, and improving insulin resistance

(1) Regulating lipid metabolism and improving hepatocellular steatosis [Evidence from preclinical animal experiments and in vitro cell experiments]: ① Inhibiting hepatic lipid synthesis at the source and reducing endogenous lipid production: On the one hand, it directly blocks the rate-limiting step of hepatic cholesterol synthesis, reduces the production of mevalonate, and inhibits the biosynthesis of endogenous cholesterol in hepatocytes [13], blocking de novo cholesterol synthesis from the upstream; on the other hand, it synergistically down-regulates the expression of SREBP1, the core transcription factor of lipid synthesis, and the expression of its downstream fatty acid synthase FASN [29], and at the same time inhibits hepatocyte glucagon resistance induced by high-cholesterol diet, restores the inhibitory effect of glucagon on hepatic lipogenesis [30], reduces the synthesis of fatty acids and triglycerides in hepatocytes, and comprehensively inhibits hepatic lipogenesis. ② Promoting hepatic lipid catabolism and enhancing lipid clearance capacity: Up-regulating the expression of peroxisome proliferator-activated receptor α (PPARα) and its downstream target genes, enhancing the fatty acid β-oxidation capacity of liver mitochondria and peroxisomes, and promoting hepatic lipid catabolism [19, 20]; at the same time, it improves mitochondrial dysfunction, further enhances the efficiency of fatty acid oxidation, and reduces liver lipid accumulation [19]. ③ Blocking lipid-mediated liver pathological injury: Through the above regulation of lipid metabolism homeostasis, it reduces the cholesterol level in hepatocytes and macrophages, reduces the formation of cholesterol crystals and lipotoxic injury, and fundamentally blocks cholesterol-mediated liver pathological injury [31], and finally improves hepatocellular steatosis.

(2) Immune regulation and anti-inflammatory effects, blocking the progression of steatohepatitis [Evidence from preclinical animal experiments and in vitro cell experiments, some targets have been verified to be abnormally expressed in liver tissue of MASH patients]: The cascade amplification of chronic liver inflammation is the core driving link for the progression of simple fatty liver to steatohepatitis. Simvastatin can simultaneously regulate the function of hepatocytes and non-parenchymal liver cells, and exert a blocking effect on the whole chain of inflammation initiation, amplification and progression. The core mechanisms are as follows: ① Inhibiting the initiation injury of hepatocyte inflammation and blocking the upstream trigger of inflammation: By reducing hepatocyte lipotoxicity and oxidative stress injury, reducing the levels of liver ROS and MDA, and restoring the activities of antioxidant enzymes such as SOD and CAT [25, 32]; at the same time, it inhibits the activation of hepatocyte ALE-RAGE pathway and abnormal activation of calpain, reduces the release of pro-inflammatory mediators and damage-associated molecular patterns, and curbs the initiation of liver inflammation from the source, and this effect is independent of the lipid-lowering effect [25, 32]. ② Regulating the innate immunity of macrophages and inhibiting the core amplification effect of inflammation: Significantly reducing the infiltration of F4/80-positive macrophages in the liver, and down-regulating the expression of key pro-inflammatory genes such as Il1b, Tnfa, and Ccl2 [28]; it can restore the expression of DHCR7 in macrophages by reducing intracellular cholesterol levels, activate the DHCR7-PI3K axis, and reverse the pro-inflammatory M1 polarization of macrophages induced by cholesterol overload. The abnormal expression of this core target has been verified in the liver tissue of MASH patients [28]. ③ Regulating the adaptive immunity of T cells and blocking the continuous progression of chronic inflammation: By inhibiting cholesterol synthesis in T cells, it reduces the infiltration, activation of liver CD8 + T cells and the secretion of pro-inflammatory factors such as IFN-γ and TNF-α. CD8 + T cell depletion experiments have confirmed that this pathway is one of the key mechanisms for simvastatin to improve MASH [33]; the antigen-driven clonal expansion and exhausted phenotype of CD8 + T cells have been confirmed as the core characteristics of disease progression in liver cirrhosis tissue of MASH patients and mouse models, providing a pathological basis for this immune regulation effect [34]. ④ Synergistically blocking the downstream effects of inflammation and curbing the progression from hepatitis to fibrosis: By dual inhibition of RhoA/Rho kinase and Ras/ERK pathways, it directly inhibits the activation of hepatic stellate cells, down-regulates the expression of pro-fibrotic genes, and reduces collagen deposition [28]; at the same time, it improves liver sinusoidal endothelial function and liver microcirculation disturbance, reduces the liver adhesion and recruitment of inflammatory cells, and further blocks the inflammatory cascade amplification and disease progression [25]. Most of the above research results related to anti-inflammatory and immune regulation are derived from independent and non-homologous experimental systems.

(3) Improving insulin and glucagon resistance and correcting systemic metabolic disorders [Evidence from preclinical animal experiments, in vitro cell experiments, verified by small-sample clinical studies]: ① Improving insulin resistance: First, simvastatin reduces the lipid load in circulating and insulin target organs such as the liver, alleviates lipotoxicity, endoplasmic reticulum stress and oxidative stress caused by ectopic lipid deposition, avoids its damage to the insulin receptor and downstream signal transduction pathway, and reverses liver-specific insulin resistance mediated by hepatic steatosis from the source; at the same time, it inhibits the overexpression and release of pro-inflammatory factors, blocks the abnormal phosphorylation of insulin receptor substrate mediated by chronic low-grade inflammation, and restores the normal conduction of insulin signals; in the local microenvironment of the liver, simvastatin can not only reverse the dedifferentiated capillarization of LSECs, restore their differentiated phenotype and material exchange function, and improve the delivery efficiency of insulin to hepatocytes, but also activate the AKT/eNOS pathway to up-regulate nitric oxide production and inhibit the abnormal expression of endothelin-1 that promotes insulin resistance, and can also reverse the activation state of HSCs, restore their quiescent phenotype, improve liver microcirculation and local microenvironment, and further relieve the inhibitory effect of pathological changes on insulin sensitivity; in addition, simvastatin can be used in combination with drugs such as Ilexgenin A and metformin to synergistically enhance the improvement effect of insulin resistance through complementary targets, and the combination with Ilexgenin A will not change its own pharmacokinetic characteristics, with good overall treatment safety [18, 21, 22]. ② Reversing hepatic glucagon resistance: By inhibiting hepatic cholesterol synthesis, reducing hepatocyte cholesterol content, restoring the signal transduction function of glucagon receptor (GCGR), enhancing glucagon-induced cAMP production and hepatic glucose output, and reversing hypercholesterolemia-induced glucagon resistance [30].

(Note: The anti-fibrosis, metabolic and immune regulation mechanisms described in this section are all derived from preclinical animal and cell experiments. For the transformation effect in the real human microenvironment and the discussion of model dependence, see Sect.  6.1 of this article.)

### Evidence boundary and clinical application tips

#### Summary of core benefits and evidence level

**Table:** 

Potential Core Benefits	Evidence Level	Core Supporting Evidence
Reducing the long-term risk of decompensated liver cirrhosis and HCC in patients with MASLD complicated with T2DM	Moderate quality	Large-sample retrospective cohort study [18]
Reducing the risk of advanced liver fibrosis in patients with MASLD complicated with T2DM	Low-to-moderate quality	Cohort study with gold standard of liver biopsy [9]
Improving blood lipid profile and insulin resistance in patients with MASLD	Low quality	Small-sample single-center clinical controlled study [24]
Alleviating hepatic steatosis, inflammation and oxidative stress, and delaying the pathological progression of MASH	Low quality	Animal experiments and in vitro cell experiments, no confirmed human clinical data[1–5, 14]

#### Reasonable scenarios for clinical application and Non-extrapolable boundaries

Reasonable application scenarios: Combined with existing evidence, the clinical application of simvastatin should focus on patients with MASLD complicated with hyperlipidemia and T2DM, especially those with liver fibrosis and high risk of cardiovascular disease. This population can obtain multiple values of blood lipid regulation, cardiovascular protection and potential liver benefits from simvastatin treatment.

Non-extrapolable evidence boundaries: ① The existing evidence cannot be extrapolated to patients with decompensated cirrhosis, and there is no research data to support the efficacy and safety of simvastatin in this population; ② Cannot be extrapolated to non-obese patients with MASLD without metabolic comorbidities, and there is no evidence for clinical benefits and safety in this population; ③ The existing evidence cannot confirm that simvastatin can reverse established liver cirrhosis or severe liver fibrosis, and can only suggest its potential value in delaying the progression of fibrosis; ④ The anti-inflammatory and anti-fibrosis mechanisms in animal experiments cannot be directly equated with the therapeutic effect in humans, and over-interpretation should be vigilant; ⑤ For patients with simple hepatic steatosis without statin lipid-lowering indications, there is currently no evidence to support the routine use of simvastatin in the treatment of MASLD.

#### Clinical practice tips

For patients with MASLD combined with statin indications, patients should be fully informed of the limitations of existing evidence, and statins with proven potential liver benefits such as simvastatin can be preferentially selected to take into account the hepatoprotective effect while achieving the lipid-lowering goal; liver function and muscle enzyme levels should be monitored during medication, and the safety specifications for clinical application of statins should be followed.

At present, it is not recommended to use simvastatin as a specific therapeutic drug for MASLD in patients without lipid-lowering indications; the basic treatment of MASLD is still lifestyle intervention (diet control, exercise, weight management), and standardized management of comorbidities is the core of the whole-course management of the disease. At present, there is no sufficient evidence-based evidence to support the use of simvastatin as a specific therapeutic drug for MASLD, and it is strictly prohibited for patients without legal indications for statin lipid-lowering, which belongs to off-label drug use in this scenario without supporting evidence of benefit. Its long-term benefits on liver clinical hard endpoints and histological improvement effect still need to be further confirmed by prospective RCTs.

## Application of simvastatin in liver cirrhosis

### Anchoring of clinical pain points

Liver cirrhosis is the end-stage pathological outcome of various chronic liver injuries, which can be divided into compensated and decompensated stages according to the disease process, and there are significant differences in the core treatment pain points: the core demand of compensated cirrhosis is to delay the progression of liver fibrosis, prevent decompensation events, and reduce the risk of HCC and all-cause death, and there is currently no approved disease-modifying therapeutic drug; decompensated cirrhosis is characterized by fatal complications related to portal hypertension, the survival period of patients is significantly shortened, existing treatment methods cannot effectively improve long-term survival, and the risk of medication safety is extremely high in end-stage patients; portal hypertension is the core pathological change throughout the whole course of liver cirrhosis, and also the fundamental cause of various complications, and traditional treatments have limitations such as systemic hemodynamic effects [5].

Simvastatin can exert pleiotropic effects such as anti-liver fibrosis, improvement of portal hypertension, anti-inflammation, and multi-organ protection through multiple targets. The following is strictly stratified according to clinical scenarios, and simultaneously presents evidence-based evidence, corresponding mechanisms, and benefit-risk boundaries.

### Clinical application in liver cirrhosis

#### Evidence-based evidence and critical evaluation of application in compensated cirrhosis

The core treatment goal of compensated cirrhosis is to prevent the progression of the disease to the decompensated stage and reduce the risk of HCC and all-cause death. Existing evidence confirms that simvastatin has clear clinical benefits in this population:

(1) Reducing the risk of liver cirrhosis decompensation and all-cause mortality: A large-sample cohort study of the US Department of Veterans Affairs showed that in patients with HCV-related compensated cirrhosis, the use of statins (85% simvastatin) was significantly associated with a reduced risk of liver cirrhosis decompensation and all-cause mortality, and the benefit showed a clear dose-dependent and cumulative exposure duration-dependent manner, with each 5-month increase in statin exposure, the risk of decompensation was further reduced [35]. Another nationwide veterans cohort study also confirmed that statin exposure was significantly associated with reduced risk of liver decompensation and all-cause mortality in patients with compensated cirrhosis, and the benefit was independent of the lipid-lowering effect [36].

(2) Improving portal hypertension and preventing first variceal bleeding: A Meta-analysis in 2020 showed that statins can significantly improve the improvement rate of portal hypertension in patients with liver cirrhosis (hepatic venous pressure gradient (HVPG) decreased by > 20% from baseline or reduced to < 12mmHg), and reduce the risk of variceal bleeding by 36% [37]. A proof-of-concept RCT confirmed that simvastatin treatment for 1 month reduced the mean HVPG of patients with liver cirrhosis by 8.3%, and 32% of patients achieved clinically significant hemodynamic response, and a significant decrease in HVPG was observed regardless of whether the patients were combined with non-selective β-blockers (NSBBs), which had an additive effect with β-blockers [38].

(3) Reducing the risk of HCC: A number of large-sample retrospective studies have shown that the use of statins is significantly associated with a reduced risk of HCC in patients with chronic liver disease. In populations with HCV and HBV-related cirrhosis, statin exposure can reduce the risk of HCC by 30%~50% in a dose-dependent manner [15, 35, 36].

(4) Preventive effect on ACLF: Preclinical studies have confirmed that in rats with liver cirrhosis, simvastatin pretreatment can significantly prevent the progression of LPS-induced ACLF, reverse the aggravation of portal hypertension, hepatic microvascular dysfunction and inflammatory outbreak caused by endotoxemia, and improve the survival rate of model animals [39]. A large-sample retrospective cohort study showed that in patients with liver cirrhosis, statin exposure was significantly associated with a 38% reduction in the risk of high-grade ACLF, with a clear dose-effect relationship. Simvastatin equivalent ≥ 20 mg/d can reduce the risk of ACLF by 39%, and the longer the cumulative exposure duration, the more significant the protective effect; at the same time, the 28-day and 90-day mortality rates of ACLF patients with statin exposure were also significantly lower than those without statin exposure [40].

(Note: The above potential clinical benefits of preventing decompensation, reducing portal pressure, preventing HCC and ACLF in the compensated stage are mainly based on retrospective observational data or surrogate endpoint indicators, lacking prospective confirmation. For detailed evidence defects, see Sect.  6.1 of this article.)

Clinical application tips: It is not recommended to use simvastatin for the intervention of established ACLF; for patients with cirrhosis at high risk of ACLF, the existing evidence only suggests potential preventive value, and individualized decision-making is required after full assessment of the benefit-risk ratio, which cannot be used as a routine preventive measure.

#### Evidence-based evidence and critical evaluation of application in decompensated cirrhosis

The liver reserve function of patients with decompensated cirrhosis is significantly reduced. The clinical evidence of simvastatin in this population has significant heterogeneity of results, with coexistence of positive benefit conclusions and negative confirmatory results. It is necessary to strictly limit the population and application scenarios, conduct critical and balanced interpretation, and strictly prohibit unsubstantiated evidence extrapolation:

(1) Core negative confirmatory clinical trial results:

The international multicenter phase 3 RCT (LIVERHOPE study) included 237 patients with decompensated cirrhosis, and given simvastatin 20 mg/d combined with rifaximin treatment for 12 months on the basis of standard treatment. The results showed that the combined treatment failed to reduce the risk of acute-on-chronic liver failure (ACLF), and had no significant improvement effect on the liver transplantation/death endpoint and the incidence of various complications of liver cirrhosis, which did not support the use of simvastatin combined with rifaximin for disease-modifying therapy in patients with decompensated cirrhosis [41].

(2) Evidence of survival benefit:

The multicenter RCT (BLEPS study) included 158 patients with liver cirrhosis after variceal bleeding. On the basis of standard treatment (NSBBs + endoscopic variceal ligation), simvastatin (20 mg/d increased to 40 mg/d) was added for up to 24 months. The results showed that although simvastatin did not reduce the incidence of the primary composite endpoint (rebleeding or death), it significantly reduced all-cause mortality by 61%, and the absolute risk of liver-related death was reduced by 10.39%, and the survival benefit was mainly seen in patients with Child-Pugh grade A/B [42]. A single-center RCT published in 2024 further confirmed that on the basis of standard treatment for patients with liver cirrhosis and variceal bleeding, adding simvastatin 20 mg/d for 24 months can reduce the all-cause mortality risk of patients by 52%, significantly improve the 2-year transplant-free survival rate, reduce the absolute mortality risk by 13.4%, and the number needed to treat (NNT) is only 8 [43]. A Meta-analysis published in 2022 included 9 RCTs of simvastatin in the treatment of liver cirrhosis, and the results showed that simvastatin can significantly reduce all-cause mortality in patients with liver cirrhosis (RR = 0.46), especially the risk of fatal bleeding-related death [44].

(3) Evidence for the prevention and treatment of liver cirrhosis complications:

① Ascites and spontaneous bacterial peritonitis (SBP): A single-center RCT in 2024 showed that simvastatin can reduce the risk of new-onset/worsening ascites by 40% and the risk of SBP by 70% in patients with decompensated cirrhosis, but the study mainly included patients with Child-Pugh grade A/B after variceal bleeding, with limited evidence extrapolation [44].

② Secondary prevention of variceal bleeding: Both the BLEPS study and Meta-analysis showed that simvastatin did not significantly reduce the incidence of variceal rebleeding [42, 44]. The core reason is that patients received standardized standard secondary prevention treatment of endoscopy + β-blockers, and the space for further blood pressure reduction is limited; however, in a double-blind RCT study, simvastatin can bring additional benefits to patients with poor response to NSBBs. The study included patients with liver cirrhosis with poor acute response to propranolol. The combination of simvastatin on the basis of carvedilol can significantly increase the reduction of HVPG (2.97mmHg vs. 2.05mmHg), increase the hemodynamic response rate from 15% to 37%, and significantly reduce the increase of postprandial portal pressure [9].

③ Other complications: Existing evidence does not show that simvastatin can significantly reduce the incidence of hepatic encephalopathy and hepatorenal syndrome, only a downward trend was observed in some retrospective studies [44], and there is a lack of prospective RCT confirmation.

(4) Differences in efficacy in special populations: The benefit of simvastatin is only seen in patients with mild to moderate decompensated cirrhosis (Child-Pugh grade A/B) [45]. Patients with severe decompensated cirrhosis of Child-Pugh grade C and MELD score > 12 have no significant benefit evidence, and the safety risk increases sharply; existing studies are mainly based on populations with alcoholic and viral cirrhosis, and the evidence for populations with MASLD-related cirrhosis and female patients is relatively insufficient.

(Note: The survival benefits suggested above are mainly seen in a narrow population after variceal bleeding (such as the BLEPS study), and are limited by the sample size and the safety risk of high-dose medication, so the population extrapolation needs to be extremely cautious. See Sect.  6.1 of this article for details.)

### Corresponding core mechanisms of action

**Fig. 2:**
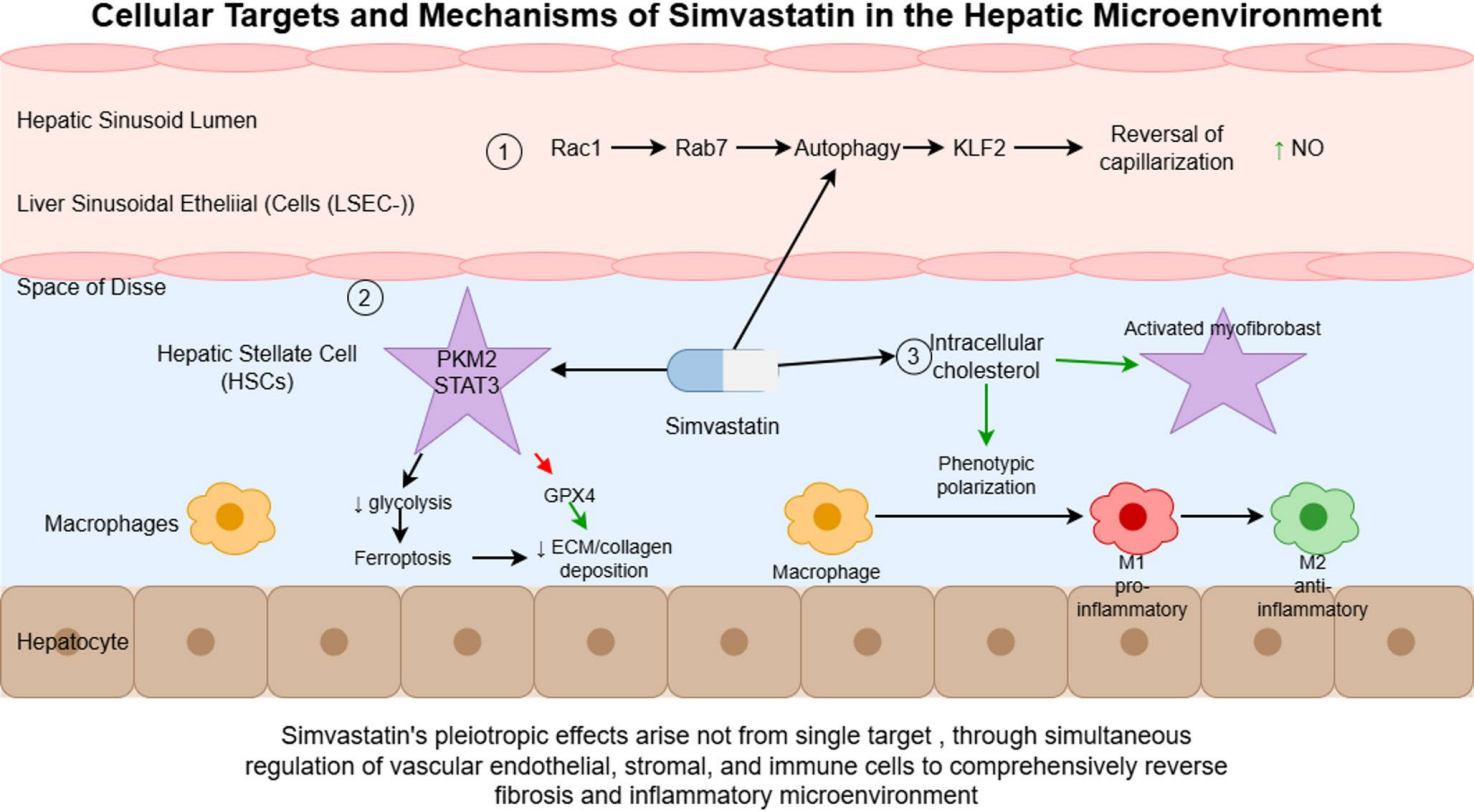
Cellular Targets and Mechanisms of Simvastatin in the Hepatic Microenvironment

This section only describes the differentiated mechanisms in the scenario of liver cirrhosis, and the basic anti-fibrosis mechanisms repeated in the MASLD chapter will not be repeated. The core includes:

(1) Supplement to the core anti-fibrosis pathway [Evidence from preclinical animal experiments and in vitro cell experiments]: ① Targeting PKM2 to regulate the metabolic reprogramming of HSCs, down-regulating the expression of PKM2 in activated HSCs, directly inhibiting aerobic glycolysis, and at the same time disrupting the PKM2/STAT3/c-MYC signal cascade, blocking the glutaminolysis of HSCs, and inhibiting the activation of HSCs and collagen synthesis from the metabolic level [46]; ② Inducing ferroptosis of activated HSCs, by inhibiting the mevalonate pathway to down-regulate the expression of GPX4, a key negative regulatory enzyme of ferroptosis in HSCs, activating the autophagy/ferritinophagy pathway, causing iron accumulation and lipid peroxidation in HSCs, and finally inducing ferroptosis in activated HSCs, and this effect has cell selectivity and has no significant effect on hepatocytes [47]; ③ Inducing deactivation and apoptosis of activated HSCs, by up-regulating the expression of KLF2, directly down-regulating the activation markers of HSCs, and at the same time activating the Nrf2 antioxidant pathway, promoting the apoptosis of activated HSCs, and reducing the number of fibrotic effector cells in the liver [48].

(2) Core mechanisms for improving portal hypertension [Evidence from preclinical animal experiments and in vitro cell experiments]: ① Improving LSEC dysfunction and reversing hepatic sinusoidal capillarization, by up-regulating the KLF2-eNOS pathway, increasing the bioavailability of NO in the liver, repairing the fenestral structure of LSECs, and restoring the material exchange and blood perfusion of the hepatic sinusoidal microcirculation [24, 48]; ② Selectively reducing intrahepatic vascular resistance, by inhibiting the contraction of HSCs and improving the endothelial function of LSECs, reducing the intrahepatic microcirculation resistance, while not reducing or even increasing hepatic blood flow and liver perfusion, and improving liver function [38, 48], forming a complementary mechanism with NSBBs; ③ Long-term treatment can reduce the formation of intrahepatic fibrous septa through anti-fibrosis effect, improve the structural disorder of the liver, reduce the structural intrahepatic resistance from the pathological level, and achieve continuous improvement of portal pressure [39].

(3) Anti-inflammatory and hepatoprotective effects [Evidence from preclinical animal experiments and in vitro cell experiments]: It can prevent the inflammatory outbreak induced by endotoxemia, reduce the infiltration of neutrophils in liver tissue and the formation of neutrophil extracellular traps (NETs) [39], inhibit the tryptophan-kynurenine pro-inflammatory pathway [49], alleviate the systemic inflammatory response accompanied by liver cirrhosis, and then reduce the risk of infection and systemic inflammation-related complications; at the same time, it can reduce the risk of multiple organ injury by improving vascular endothelial dysfunction Fig 2.

(Note: The cell regulation and portal pressure lowering mechanisms in the liver cirrhosis scenario described above are mostly from preclinical models, and lack of direct verification data in humans. See Sect.  6.1 of this article for details.)

### Clinical application tips

For patients with compensated cirrhosis complicated with dyslipidemia, the use of simvastatin can obtain potential benefits of delaying the progression of liver fibrosis, reducing the risk of portal hypertension and decompensation events while carrying out lipid-lowering treatment. Based on the recommendations of existing clinical studies, the recommended dosing regimen is 20 mg/d initial dose, taken orally before bedtime, adjusted according to efficacy and tolerance, with the maximum dose not exceeding 40 mg/d, and the safety is similar to that of the general population [50]. Individualized benefit-risk assessment must be completed before medication, liver function and creatine kinase should be routinely monitored during medication, and the safety specifications for statin medication should be followed.

For patients with mild to moderate decompensated cirrhosis (Child-Pugh grade A/B) with a history of variceal bleeding, on the basis of standard secondary prevention treatment, after comprehensive assessment of the benefit-risk ratio and full disclosure of the limitations of evidence and the attribute of off-label drug use, simvastatin 20 mg/d can be carefully added to improve the survival prognosis [42, 43], and the dose of 40 mg/d and above is strictly prohibited.

For patients with Child-Pugh grade B without clear lipid-lowering indications but at high risk of ascites and SBP, it is necessary to conduct individualized and prudent use of 20 mg/d dose after comprehensive assessment and full disclosure of the limitations of evidence and risks, and routine use is not recommended.

For patients with severe decompensated cirrhosis (Child-Pugh grade C, MELD score > 12 points), routine use of simvastatin is not recommended. Only when there are strong cardiovascular indications, it can be used very cautiously at an extremely low dose of ≤ 10 mg/d under close monitoring, and doses above 20 mg/d are strictly prohibited [50].

Routine use of simvastatin for disease-modifying therapy and prevention of ACLF and non-bleeding-related liver cirrhosis complications in patients with decompensated cirrhosis is not recommended, and existing evidence cannot support this clinical application [41].

It is not recommended to use simvastatin for the intervention of established ACLF; for patients with cirrhosis at high risk of ACLF, the existing evidence only suggests potential preventive value, and individualized decision-making is required after full assessment of the benefit-risk ratio, which cannot be used as a routine preventive measure.

### Research progress of novel targeted delivery systems

The application of simvastatin in the treatment of liver cirrhosis is limited by dose-dependent muscle and liver toxicity, with a narrow therapeutic window. Nano-targeted delivery technology can significantly improve its liver targeting, reduce systemic exposure, achieve efficacy enhancement and toxicity reduction. All existing studies are in the preclinical animal experiment stage, and there is no human clinical trial data. The core progress is as follows:

Pluronic-based polymeric micelle delivery system: Simvastatin-loaded polymeric micelles prepared based on Pluronic F127/F108 have a particle size of about 20 nm. After intravenous injection, about 50% of the drug is distributed in the liver, and there is almost no distribution in the muscle; in the BDL liver cirrhosis rat model, the micelle preparation at 5 mg/kg can significantly reduce portal pressure without the toxicity such as weight loss and elevated liver enzymes caused by oral simvastatin, significantly expanding the therapeutic window [51].

Hepatic sinusoidal barrier repair-type nano-preparations: Hyaluronic acid-modified simvastatin-loaded lipid nanoparticles can accurately target capillarized LSECs, efficiently repair the fenestral structure of hepatic sinuses, reverse hepatic sinusoidal capillarization, break the first pathological barrier of liver fibrosis, and at the same time improve the intrahepatic penetration efficiency of subsequent anti-fibrotic drugs [52].

Clinical translation tips: All targeted preparations are in the preclinical research stage, and their targeting efficiency, safety and clinical benefits in humans have not been verified. They cannot be used in clinical practice at present, and are an important direction for future translational research.

### Evidence summary and clinical boundaries

Clarity of benefit population: Simvastatin has the clearest benefit in the population with compensated cirrhosis, with relatively sufficient evidence [35–37]; patients with mild to moderate decompensated stage (Child-Pugh grade A/B) have potential survival benefits, but the research results are heterogeneous [42, 43]; patients with severe decompensated stage have limited benefits and extremely high safety risks, which is a clearly not recommended population [45, 50].

Core evidence defects: Lack of large-sample multicenter RCTs with long-term clinical hard endpoints as the core, existing research results are heterogeneous, with coexistence of negative and positive conclusions [41, 42]; the pharmacokinetic data of patients with decompensated cirrhosis is completely missing, and it is impossible to formulate an accurate individualized dose regimen; the evidence for special populations (women, MASLD-related cirrhosis, elderly patients) is seriously insufficient.

Core safety boundary: Adverse reactions are significantly dose-dependent. The dose within 40 mg/d in patients with compensated stage has good safety, only the dose of 20 mg/d in patients with decompensated stage has controllable safety, and the dose of 40 mg/d and above will significantly increase the risk of muscle toxicity, and routine use is strictly prohibited [50, 53, 54].

## Application and mechanism of action of simvastatin in the prevention and treatment of hepatocellular carcinoma

### Anchoring of clinical pain points

The current clinical diagnosis and treatment of HCC face three core pain points: First, the primary prevention methods for high-risk groups of chronic liver disease are limited, the existing etiological treatment cannot completely eliminate the risk of HCC carcinogenesis, and there is a lack of targeted prevention strategies; second, the 5-year tumor recurrence rate after radical surgical resection of HCC is 50%~70%, and the overall 5-year recurrence rate of radical ablation therapy is 40%~60%. At present, there is no approved effective adjuvant therapy regimen to reduce the risk of postoperative recurrence [5]; third, the efficacy of systemic therapy for advanced HCC is limited, the objective response rate of targeted and immunotherapy is low, and drug resistance and immunosuppressive microenvironment are the core reasons for treatment failure [10, 11, 55].

As a classic lipid-lowering drug, simvastatin has good safety after decades of clinical application. A large number of preclinical studies and epidemiological studies have shown that it has potential application value in the primary prevention of HCC, secondary prevention of postoperative recurrence, and combined treatment of advanced stage.

### Primary prevention of HCC (prevention of new onset carcinogenesis in high-risk populations)

#### Clinical evidence-based evidence and critical evaluation

The existing clinical evidence on simvastatin and the risk of HCC incidence mainly comes from retrospective observational studies and Meta-analyses, and there is no confirmatory evidence from large-sample prospective RCTs.

Core evidence-based results: A Meta-analysis including 11 observational studies covering more than 2.37 million people showed that compared with the population not exposed to statins, the incidence of HCC in the simvastatin exposure group was significantly reduced, with a pooled odds ratio (OR) of 0.59 (95%CI 0.51–0.67, *P* < 0.001), and this effect was more significant in the populations of Eastern countries (China, South Korea); at the same time, overall exposure to lipophilic statins can reduce the risk of liver cancer by 46% (OR = 0.54, 95%CI 0.48–0.60), and simvastatin is one of the core statin types showing significant preventive effects [56]. A number of clinical cohort studies have also suggested that in high-risk groups of HCC such as chronic viral hepatitis and T2DM, the use of simvastatin is significantly associated with a reduced risk of HCC incidence and all-cause mortality [15, 35].

(Note: Most of the above primary prevention evidence comes from retrospective cohort analysis, there are confounding factors such as indication bias that cannot be completely eliminated, and the absolute causal relationship cannot be established. See Sect.  6.1 of this article for details.)

#### Corresponding core mechanisms of action

The mechanisms in this section all correspond to the clinical benefits of blocking malignant transformation of hepatocytes and reducing the risk of carcinogenesis in primary prevention. All effects are verified by in vitro cell experiments and animal models. The relevant molecular targets and subtype-specific effects are only exploratory hypotheses, and there is no prospective clinical trial data of simvastatin intervention in humans, which cannot be directly deduced into clinical preventive effects or used as the basis for clinical medication. The details are as follows:

(1) Improving chronic inflammation and the precancerous microenvironment of liver fibrosis [Evidence from preclinical animal experiments]: Liver fibrosis is the core precancerous pathological link of HCC [8]. Simvastatin can activate the KLF2-NO signaling pathway of LSECs, reverse the capillarization of LSECs, mediate the deactivation of activated HSCs, reduce collagen deposition, and alleviate the progression of liver fibrosis [48]; at the same time, it can down-regulate the expression of TGF-β1 in a dose-dependent manner, block its mediated abnormal proliferation and malignant transformation of hepatocytes [57]. In the rat HCC model induced by DEN combined with TAA, it can significantly reduce the liver necrosis inflammation score, reduce the deposition of collagen fibers in liver tissue, and improve the pathological progression of chronic liver injury [58].

(2) Inhibiting the malignant transformation of hepatocytes and blocking the activation of carcinogenic pathways [Evidence from in vitro cell experiments and preclinical animal experiments]: ① For HBV infection-related carcinogenesis, it can dose-dependently inhibit the accumulation of cholesterol in hepatocytes mediated by HBV-miR-3, block its induced increase in hepatocyte viability and enhancement of malignant phenotype, and can significantly reverse this carcinogenic effect at a concentration of ≥ 1µM [59]; ② For the malignant transformation of hepatocytes induced by environmental carcinogens, it can completely reverse the abnormal proliferation of hepatocytes induced by environmental pollutant HCB, block its mediated cholesterol metabolism disorder, and eliminate the driving factors of malignant transformation induced by carcinogens [57]; ③ By inhibiting the mevalonate pathway, it reduces the isoprenylation modification of oncogenic proteins such as Ras and Rho, blocks their mediated pro-proliferation and pro-transformation signals, and inhibits the process of malignant transformation of hepatocytes from the source [57].

#### Clinical application tips

The existing evidence can only confirm the correlation between the use of simvastatin and the reduced risk of HCC incidence, but cannot confirm the causal effect [56]. Therefore, it is not recommended that high-risk groups of HCC without lipid-lowering indications routinely use simvastatin for chemoprevention of HCC. For high-risk populations of chronic liver disease with statin indications for lipid lowering, simvastatin can be preferentially selected to take into account the potential benefit of HCC prevention while achieving the lipid-lowering goal, and patients should be fully informed of the limitations of existing evidence.

### Secondary prevention of recurrence after radical resection of HCC

#### Clinical evidence-based evidence and critical evaluation

The existing clinical evidence of simvastatin in the secondary prevention of recurrence after radical resection of HCC is extremely limited, only from small-sample retrospective clinical studies, without confirmatory data from prospective RCTs, which cannot support the routine application of simvastatin in patients after radical resection of HCC. It is not recommended to routinely use simvastatin for the secondary prevention of postoperative recurrence of HCC outside clinical trials.

Existing clinical research data: A retrospective cohort study including 275 HCC patients who underwent R0 radical resection showed that in 29 patients who continued to take statins (14% of which were simvastatin) before and after surgery, there was no statistically significant difference in overall survival (OS) and recurrence-free survival (RFS) between the statin intake group and the non-intake group in the full cohort analysis; however, after excluding users of hydrophilic pravastatin, the RFS of the lipophilic statin (including simvastatin) intake group was significantly better than that of the non-statin intake group (*P* = 0.038), suggesting that lipophilic statins such as simvastatin may have potential benefits in improving the risk of postoperative recurrence of HCC [60].

(Note: The potential benefit of secondary prevention is only based on a single-center, very small-sample retrospective subgroup analysis, with an extremely low level of evidence. See Sect.  6.1 of this article for details.)

#### Clinical application tips

The existing evidence cannot support the routine application of simvastatin in patients after radical resection of HCC, and it is not recommended to routinely use simvastatin for the secondary prevention of postoperative recurrence of HCC outside clinical trials. For patients with statin lipid-lowering indications after surgery, it can be used according to conventional indications, and there is no need to adjust the medication due to the history of HCC. At the same time, patients should be informed that the existing evidence cannot confirm its preventive effect on postoperative recurrence.

### Combined adjuvant application in systemic therapy for advanced HCC

#### Core evidence from preclinical studies

(1) Combined targeted therapy (TKI): Reversing drug resistance and synergistically enhancing efficacy [Evidence from in vitro cell experiments and animal models]: Simvastatin can significantly enhance the anti-tumor effect of HCC targeted drugs and reverse acquired drug resistance. In the sorafenib-resistant HCC cell model and PDX drug-resistant model, simvastatin can block the aerobic glycolysis of drug-resistant cells by inhibiting the HIF-1α/PPAR-γ/PKM2 axis, reducing the half maximal inhibitory concentration (IC50) of sorafenib from 16.33µM to 4.47µM. The combination of the two has a synergistic anti-tumor effect (combination index CI = 0.722), and can completely inhibit tumor growth in the in vivo drug-resistant model [61]. At the same time, it can block the enrichment and drug resistance of liver cancer stem cells induced by sorafenib and lenvatinib by inhibiting the CASP3/SREBP2-mediated cholesterol synthesis pathway. In HCC patient-derived organoids and drug-resistant PDTX models, the combined treatment can significantly inhibit tumor growth, and the effect is greatly improved compared with monotherapy [62]. In addition, the combination of simvastatin with EGFR-TKI, RAF inhibitors, and MEK inhibitors has shown synergistic anti-proliferative effects in a variety of HCC cell lines, which can simultaneously block the EGFR pathway and YAP oncogenic signal, and enhance the inhibitory effect on tumor cell survival [63].

(2) Combined cellular immunotherapy: Improving T cell function and synergistic killing [Evidence from animal models]: In the humanized HCC PDX model, simvastatin can regulate the reprogramming of tumor lipid metabolism by targeted inhibition of HMGCR, and at the same time up-regulate the cholesterol level of activated T cells, improve the function of effector T cells, and exert a synergistic anti-HCC effect with CAR-T cells. The combination of the two can completely inhibit the growth of HCC tumors, which is significantly better than T cell monotherapy [10]. In addition, simvastatin can activate the TCR signaling pathway of CD8 + T cells, enhance the activation, proliferation and antigen-specific killing ability of CD8 + T cells, and down-regulate the expression of T cell exhaustion markers [64], which provides a mechanistic support for the combination of adoptive cell therapy.

(3) Combined immune checkpoint inhibitors: Remodeling the tumor microenvironment and reversing immunosuppression [Evidence from animal models]: The simvastatin targeted delivery system can up-regulate the expression of CXCL16 in LSECs, recruit and activate NKT cells, and reverse the immunosuppressive microenvironment of HCC. In the mouse model of advanced HCC, the combination of simvastatin targeted nanoparticles and anti-PD-L1 antibody can significantly inhibit tumor progression, completely inhibit lung metastasis, achieve a significant prolongation of survival compared with monotherapy, and the two show clear synergistic therapeutic potential [65].

#### Clinical application tips

The existing conclusions of synergistic efficacy enhancement and drug resistance reversal are all from in vitro cell experiments and animal models, which are hypothesis-generating research results. Their effectiveness, safety, optimal administration dose and combined regimen in humans are not clear. It is not recommended to use simvastatin combined with targeted and immunotherapy for the routine clinical treatment of patients with advanced HCC outside clinical trials.

### Potential mechanisms of simvastatin against HCC: exploratory hypotheses based on preclinical models

(1) Regulating cell cycle, inducing apoptosis and programmed death of tumor cells [Verified in a variety of HCC cell lines and in vivo xenograft tumor models]: Simvastatin can up-regulate the expression of cyclin-dependent kinase inhibitors p21 and p27 by activating the AMPK pathway and inhibiting the Jak1/Jak2-STAT3 signal axis, arrest the HCC cell cycle in the G0/G1 phase, and inhibit cell division and proliferation [55]; at the same time, it can induce apoptosis of HCC cells by activating the caspase-dependent mitochondrial apoptosis pathway, up-regulate the expression of pro-apoptotic proteins, and down-regulate the level of anti-apoptotic protein Bcl-2 [58]. This effect has a clear dose-dependent and time-dependent manner, and is completely dependent on the inhibition of HMGCR [55]. Its high lipophilicity enables it to efficiently enter and accumulate in HCC cells, which is an important basis for exerting cytotoxic and pro-apoptotic effects, while hydrophilic statins have no such significant effect [66, 67].

(2) Inhibiting the mevalonate pathway and correcting the disorder of cholesterol metabolism in tumor cells [Verified in in vitro cell experiments and animal models]: Abnormal activation of the mevalonate pathway is the core driving factor for the occurrence and development, stemness maintenance, and drug resistance of HCC [62, 63, 68]. By competitively inhibiting HMGCR, simvastatin not only reduces de novo synthesis of cholesterol, but also reduces the production of downstream products farnesyl pyrophosphate (FPP) and geranylgeranyl pyrophosphate (GGPP), and blocks the isoprenylation modification and pro-cancer signal transduction of small GTPases such as Ras and Rho [55, 57]; at the same time, it can correct the reprogramming of cholesterol metabolism in HCC cells, and block the positive feedback activation of the cholesterol synthesis pathway mediated by SREBP2 [62], and reverse the abnormal hyperactivity of cholesterol synthesis mediated by down-regulation of LDLR [69].

(3) Inhibiting the stemness maintenance of liver cancer stem cells [Verified in in vitro cell experiments and animal models]: Liver cancer stem cells (CSCs) are the root cause of tumor recurrence, metastasis and drug resistance [62, 70]. Simvastatin can significantly reduce the spheroidization ability of HCC cells, down-regulate the expression of stemness markers such as CD133, EpCAM, and NANOG, reduce the proportion of CSCs population, and inhibit the in vivo tumorigenic ability and tumor initiation frequency of HCC cells by inhibiting HMGCR[76]; the core mechanism is to block the activation of the downstream SHH signaling pathway by inhibiting cholesterol synthesis [70], which is the core downstream effector pathway of HMGCR regulating HCC stemness [62].

(4) Reversing targeted therapy resistance and regulating tumor metabolic reprogramming [Verified in drug-resistant cell models and PDX models]: It can reverse the glycolytic phenotype of sorafenib-resistant cells by inhibiting aerobic glycolysis and down-regulating the expression of key enzymes of the Warburg effect [61, 71]; at the same time, it can re-sensitize drug-resistant HCC cells to targeted drugs by inhibiting drug resistance-related pathways such as the HIF-1α/PPAR-γ/PKM2 signaling axis and YAP/STAT3 [61, 63]; in addition, it can differentially regulate lipid metabolism between tumor cells and immune cells, improve the function of effector T cells while inhibiting the metabolism of tumor cells [33, 64], providing a basis for combined immunotherapy.

(5) Inhibiting epithelial-mesenchymal transition (EMT) and tumor metastasis, remodeling the tumor microenvironment [Verified in in vitro cell experiments and animal models]: It can up-regulate the expression of epithelial marker E-cadherin, down-regulate mesenchymal markers, reverse the EMT process of HCC cells, and significantly inhibit cell migration and invasion ability by regulating the PTEN/AKT signal axis [72]; at the same time, it can alleviate tumor interstitial fibrosis, improve the penetration of drugs in tumor tissue, reshape the immunosuppressive tumor microenvironment, and enhance the tumor infiltration and killing activity of immune cells [65].

Supplementary note: The differential effects on HCC molecular subtypes such as ASPP2 deficiency, low LDLR expression, and high HMGCR expression are only preclinical exploratory hypotheses, and their predictive value has not been verified in the clinical cohort of HCC patients, and cannot be used to guide clinical medication decision-making.

(Note: The molecular mechanisms of anti-HCC, the regulation of targeted pathways, and the efficacy-enhancing conclusions of advanced combined treatment described in this section are currently exploratory hypotheses based on in vitro and animal models, and have not been confirmed in human clinical trials. For the transformation limitations, see Sect.  6.1 of this article.)

### Tips for future research directions

Existing studies suggest that the anti-HCC effect of simvastatin has population and tumor subtype specificity, and the effect of lipophilic statins is significantly better than that of hydrophilic statins [56, 66, 67]. It may have more significant potential benefits in patients with HBV-related HCC, HCC complicated with hypercholesterolemia, and HCC with high HMGCR expression, but this conclusion only comes from preclinical studies and still needs to be verified by clinical studies. In the future, it is urgent to give priority to carrying out large-sample, prospective, randomized controlled clinical trials to clarify the clinical benefits and risks of simvastatin in the primary prevention of HCC, and gradually fill the gaps in clinical research on postoperative secondary prevention and advanced combined treatment.

## Safety and clinical medication specifications of simvastatin in liver diseases

This specification is formulated based on clinical studies, RCTs, authoritative guidelines, drug instructions and basic experimental evidence related to MASLD, liver cirrhosis and HCC, integrates all medication-related content in the full text, centrally clarifies the core content such as medication indications, contraindications, dose regimens, and safety management of simvastatin in different liver disease scenarios, and clearly marks the medication scenarios without evidence support, completely solving the problem of repeated content across chapters, and improving clinical practicability through structured tables.

### Clinical indications and applicable populations

This section clarifies the evidence-based supported application scenarios of simvastatin in different liver diseases, and marks the off-indication scenarios without evidence support.

#### Metabolic dysfunction-associated steatotic liver disease (MASLD)

(1) Patients with MASLD complicated with hyperlipidemia, especially those with T2DM and high risk of cardiovascular disease, for blood lipid management, while having potential benefits of improving hepatic steatosis and inflammation [18, 20, 21].

(2) Patients with T2DM complicated with MASH and liver fibrosis, which can be used to delay the progression of liver fibrosis and reduce the long-term risk of liver cirrhosis and HCC [15, 16].

#### Liver cirrhosis

(1) Patients with compensated cirrhosis complicated with dyslipidemia, for lipid-lowering treatment, while taking into account the potential benefits of delaying the progression of liver fibrosis, reducing the risk of portal hypertension and decompensation events [35, 36, 38].

(2) Patients with liver cirrhosis complicated with portal hypertension and high-risk esophagogastric varices, with poor hemodynamic response to traditional NSBBs, can be used in combination with carvedilol to enhance the effect of reducing portal pressure [9].

(3) Patients with mild to moderate decompensated cirrhosis (Child-Pugh grade A/B) with a history of variceal bleeding, can be carefully added to improve the survival prognosis on the basis of standard secondary prevention treatment [42, 43].

##### Special Statement

For patients with decompensated cirrhosis without statin lipid-lowering indications, the routine use of simvastatin for disease-modifying therapy, prevention of ACLF or non-bleeding-related liver cirrhosis complications has no evidence-based medical support (the core basis is the negative results of the international multicenter phase 3 LIVERHOPE study), and clinical application is not recommended.

#### HCC-related scenarios

(1) It can only be used for high-risk groups of HCC with underlying liver disease, and takes into account the potential primary prevention benefit on the premise of having statin lipid-lowering indications [15, 56].

(2) There is no clinical evidence to support the use of simvastatin in the treatment of HCC, including postoperative recurrence prevention [60] and advanced anti-tumor treatment [10, 61], and off-indication medication is strictly prohibited.

### Medication contraindications and cautious use populations

#### Absolute contraindications

(1) Hypersensitivity to any component of simvastatin; (2) Active liver disease: including acute viral hepatitis, active phase of autoimmune hepatitis, and unexplained persistent elevation of transaminases (ALT/AST > 3 times the upper limit of normal (ULN)); (3) Concomitant use of potent CYP3A4 inhibitors: such as itraconazole, ketoconazole, erythromycin, clarithromycin, ritonavir, gemfibrozil, etc. These drugs can lead to a more than 10-fold increase in simvastatin exposure, greatly increasing the risk of poisoning; (4) Pregnancy and lactation: Simvastatin interferes with cholesterol synthesis and may affect fetal development, and is classified as a pregnancy category X drug [73].

#### Cautious use populations and risk stratification

(1) Patients with advanced liver failure (Child-Pugh grade C): Due to hemodynamic changes and loss of liver enzyme activity in such patients, the systemic exposure of simvastatin increases geometrically. In the BLEPS study, the only 2 cases of rhabdomyolysis occurred in the Child-Pugh grade C population [42].

(2) Patients with MELD score > 12 points: MELD score is considered a sensitive indicator for evaluating the safety of simvastatin. A score exceeding 12 points indicates an extremely high risk of muscle toxicity, and even a 20 mg dose requires caution [54, 74].

(3) Chinese and East Asian populations: Due to genetic polymorphisms such as SLCO1B1 (encoding the OATP1B1 transporter), the tolerated dose of statins in the Chinese population is usually lower than that in Caucasians, and low-to-moderate intensity should be the main initial application [75].

(4) Heavy drinkers and long-term alcohol abusers: Alcohol not only induces changes in liver enzymes, but also may produce synergistic hepatotoxicity with simvastatin.

(5) Patients with electrolyte disorders or severe infection: Such a state is easy to induce acute kidney injury, and the use of simvastatin will increase the risk of secondary renal failure caused by rhabdomyolysis.

### Suggested medication reference based on existing clinical research evidence

This part formulates a standardized dosing regimen for different scenarios based on clinical research evidence, and clarifies the principles of dose selection and adjustment. The core recommendations are shown in the Table 2 below.

**Table 2:** Comprehensive Guidance for Clinical Application and Safety Monitoring of Simvastatin in Different Liver Disease Populations (Recommendations Based on Existing Clinical Studies)

Target Population and Liver Function Status	Recommended Dose and Maximum Limit	Potential Clinical Benefits (Evidence Level)	Core Toxicity Risk Characteristics	Clinical Monitoring and Intervention Path
MASLD complicated with hyperlipidemia or T2DM (basically normal liver function)	Conventional dose: 20 mg/d, taken orally before bedtime.< br> Limit: the same as the general cardiovascular disease population.	Regulating blood lipids and cardiovascular protection; reducing the long-term risk of liver cirrhosis and HCC.< br> (Moderate quality)	Muscle/liver toxicity: the safety is similar to that of the general population, without additional risks.	Routine monitoring: recheck 1 month, 3 months, 6 months after medication, and recheck liver function and muscle enzymes every 6–12 months for long-term medication.< br > Tip: The protective effect requires long-term accumulation (≥ 245 days/year).
Portal hypertension with poor response to NSBBs (compensated stage)	Initial: 20 mg/d.< br> Adjustment: if tolerated after 2 weeks, can be increased to 40 mg/d.	Further reduce HVPG and significantly improve the hemodynamic response rate.< br> (Low-to-moderate quality)	Synergistic risk: the combination with carvedilol has good safety, no superimposed adverse effects on systemic hemodynamics.	Routine monitoring: monitor blood pressure, heart rate and routine liver and kidney function.
Compensated cirrhosis (Child-Pugh grade A)	Initial: 20 mg/d.< br> Limit: maximum no more than 40 mg/d.	Delaying the progression of liver fibrosis, reducing the risk of decompensation events and all-cause mortality.< br> (Low-to-moderate quality)	Muscle/liver toxicity: no report of rhabdomyolysis in this population; does not increase the risk of drug-induced liver injury.	Routine monitoring: complete individualized benefit assessment before medication, routine recheck is sufficient.
Mild to moderate decompensated cirrhosis (Child-Pugh grade A/B and MELD ≤ 12 points)	Maximum limit: strictly limited to 20 mg/d.< br> Medication red line: 40 mg/d is strictly prohibited.	Only confirmed in patients with a history of variceal bleeding to reduce the risk of death and ascites complications.< br> (Low-to-moderate quality)	High-risk warning: 40 mg/d will lead to a sharp rise in the risk of rhabdomyolysis (40 times higher than that of the general population); 20 mg/d is relatively safe but still has a very low risk.	Intensive monitoring: the frequency is shortened to recheck every 1–3 months; if unexplained myalgia occurs, CK must be checked immediately.< br > Red line: routine use is not recommended for non-bleeding patients without lipid-lowering indications.
Severe decompensated cirrhosis (Child-Pugh grade C or MELD > 12 points)	Routine contraindication.< br > Use ≤ 10 mg/d under close monitoring only in a very small number of cases with strong cardiovascular indications.	No clear evidence of liver disease benefits.< br> (Very low quality)	Extremely high risk: loss of liver enzyme activity leads to geometric increase in systemic exposure, and MELD > 12 is a strong predictor of severe muscle toxicity.	Close monitoring: doses of 20 mg/d and above are absolutely prohibited, and close dynamic monitoring is required during the whole course of medication.
【Global Mandatory Drug Withdrawal and Intervention Indications】	-	-	Mild: myalgia or mild elevation of CK.< br> Moderate to severe: CK > 3 times ULN or myonecrosis occurs.< br> Severe: confirmed rhabdomyolysis.	Mild: reduce the dose to 10 mg/d, monitor weekly.< br> Moderate to severe: suspend medication, and try titration again after the indicators return to normal (2–7 weeks).< br> Severe: permanent discontinuation of statins.

### Core adverse reactions and clinical management specifications

#### Muscle-related adverse reactions

##### Clinical manifestations and classification

(1) Myalgia and Asthenia: Myalgia or fatigue is the most common side effect of simvastatin, usually manifested as symmetrical muscle soreness or “flu-like” symptoms, and the patient’s creatine kinase (CK) level may be normal at this time [44, 54].

(2) Myopathy and Myonecrosis: Accompanied by muscle weakness or abnormal elevation of CK level. Myonecrosis is classified into mild (> 3 times ULN), moderate (> 10 times ULN) and severe (≥ 50 times ULN) according to the level of CK elevation [54].

(3) Rhabdomyolysis: The most severe muscle toxicity, manifested as severe myonecrosis accompanied by myoglobinuria or renal failure [54].

##### Incidence and correlation with dose/disease condition

(1) Incidence: In patients with decompensated cirrhosis, the incidence of muscle injury (MI) caused by simvastatin is relatively high. A study showed that 36.7% of patients developed muscle injury, of which 23.4% were myalgia and 13.3% were myonecrosis [54].

(2) High dose (40 mg/d) is very easy to induce rhabdomyolysis: 40 mg/d of simvastatin is extremely dangerous in patients with decompensated cirrhosis. A systematic review showed that the incidence of rhabdomyolysis was 2% in patients taking 40 mg/d, which was 40 times higher than that in the population without liver cirrhosis, and all occurred in patients with Child-Pugh grade B and C with poor liver function [50]. In the LIVERHOPE-SAFETY phase 2 trial, as high as 19% (3/16) of patients taking 40 mg/d developed rhabdomyolysis [53].

(3) Low dose (20 mg/d) has relatively high safety but still has risks: The muscle adverse reactions of 20 mg/d dose are significantly less than those of 40 mg/d [50, 53]. However, in the latest large-sample phase 3 clinical trial of LIVERHOPE, even with the dose of 20 mg/d, 2.6% (3/117) of patients still developed rhabdomyolysis [41].

##### Management principles of muscle-related adverse reactions

(1) Dose Reduction and Drug Withdrawal Strategies.

Mild myalgia or myopathy: When the patient only has myalgia or mild myopathy (normal or slightly elevated CK), the dose of simvastatin should be reduced to 10 mg/d.

Myositis or myonecrosis: Once myositis or moderate to severe myonecrosis occurs (significantly elevated CK), simvastatin should be temporarily discontinued.

Rhabdomyolysis or severe muscle toxicity: If the patient meets the clinical criteria for rhabdomyolysis, or has severe treatment-related muscle toxicity reactions, simvastatin must be permanently discontinued, and corresponding basic life support and treatment should be carried out [41, 53, 54].

(2) Dose Adjustment and Re-medication.

For patients who temporarily stop taking the drug or reduce the dose due to muscle injury, clinical and biochemical indicator monitoring should be carried out weekly. If the biochemical indicators (such as CK level) return to normal and the muscle symptoms completely disappear (usually takes 2 to 7 weeks), you can try to start taking simvastatin again from a low dose of 10 mg/d. After restarting, the dose can be gradually titrated and increased according to the patient’s tolerance to find the maximum dose that the patient can tolerate [54].

(3) Prevention and Clinical Medication Recommendations.

Prohibition of high doses: It is strongly recommended to avoid the use of 40 mg/d simvastatin in patients with decompensated cirrhosis.

Recommended initial dose: In clinical application or research for decompensated cirrhosis, it is recommended to limit the dose of simvastatin to 20 mg/d or lower to ensure medication safety [50, 53].

Vigilance for drug interactions: Attention should be paid to the risk of combined medication when using simvastatin. For example, it should be avoided to be used in combination with potent CYP3A4 inhibitors; in addition, studies have pointed out that in some populations (such as Asians), the combination of simvastatin with niacin and other drugs will significantly increase the risk of myopathy [44]. The patient’s muscle symptoms and CK levels should be closely monitored during use.

#### Liver-related adverse reactions

(1) Characteristics of adverse reactions: Existing evidence has not confirmed that simvastatin can cause drug-induced liver injury in patients with chronic liver disease. A number of RCTs have shown that simvastatin treatment will not lead to persistent elevation of transaminases and bilirubin in patients with liver cirrhosis and MASLD, and even improve the liver function indicators of patients [42, 54]; only at high doses, a small number of patients have transient transaminase elevation, which can recover quickly after drug withdrawal [53]. The FDA has canceled the requirement for routine continuous monitoring of liver enzymes during statin treatment, and only recommends baseline testing and testing when clinically necessary.

(2) Clinical management principles:

Baseline liver function should be tested before medication to distinguish whether the elevation of liver enzymes is caused by underlying liver disease or drug-related injury; if abnormal transaminases occur, first exclude risk factors and judge the correlation with statins. If ALT/AST rises to ≥ 3×ULN combined with elevated total bilirubin, the dose should be reduced or the drug should be discontinued as appropriate. If transaminases are elevated but ALT/AST < 3×ULN, recheck transaminases after 2 ~ 4 weeks [76].

#### Other adverse reactions

(1) Risk of new-onset diabetes: Statins may slightly increase the risk of diabetes (OR about 1.1), especially for patients with MASLD who have a background of metabolic syndrome. However, considering its comprehensive benefits in MASLD (cardiovascular benefits and liver benefits), it is not recommended to stop statins due to concerns about blood sugar [75, 77].

(2) Nervous system effects: A very small number of patients report changes in cognitive function (such as amnesia, confusion), which can be recovered after drug withdrawal. Because patients with liver cirrhosis are prone to hepatic encephalopathy, clinicians need to carefully differentiate when assessing neuropsychiatric symptoms [73].

### Drug-drug interactions and safety of combined medication

**Fig. 3:**
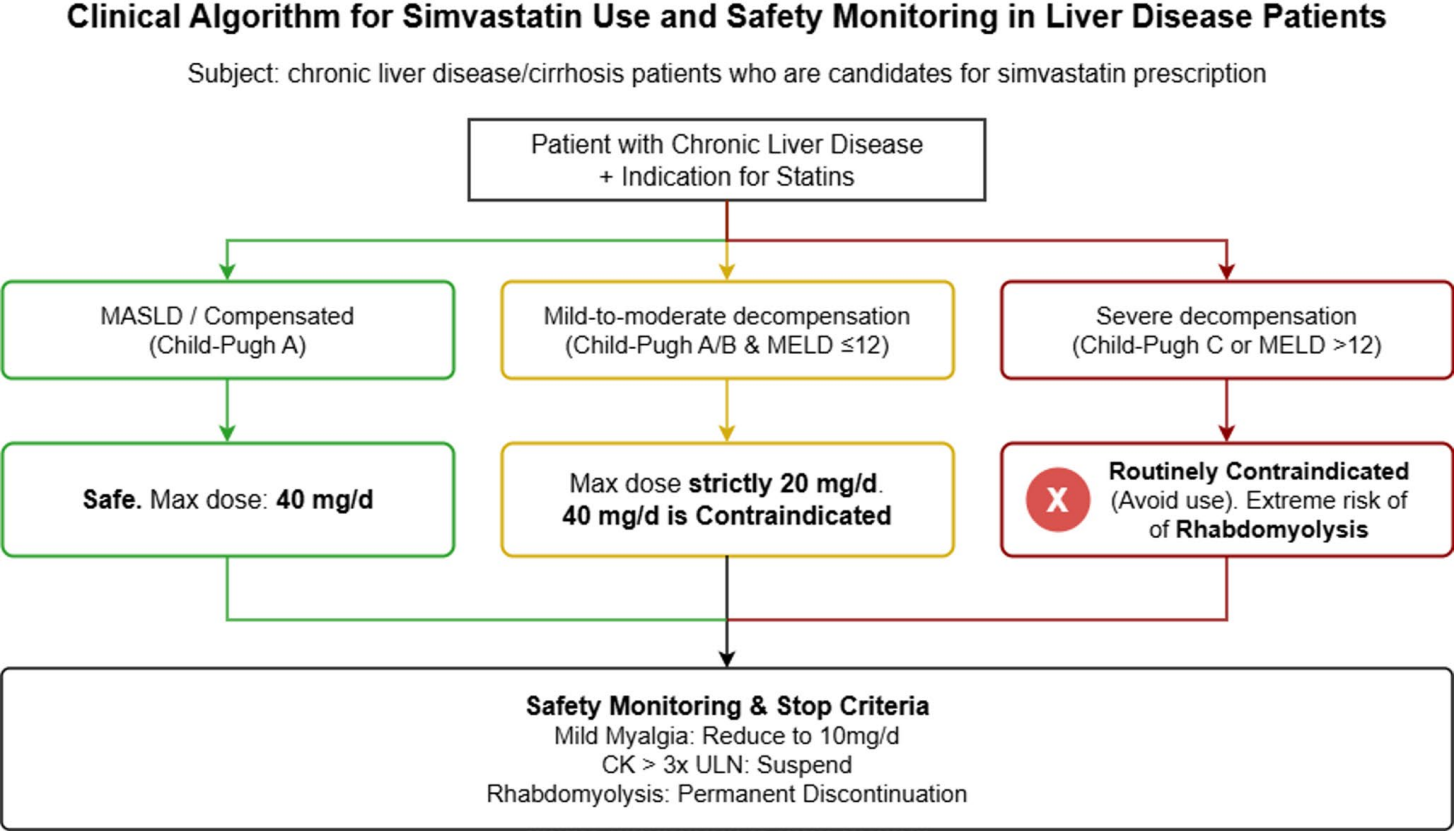
Clinical Algorithm for Simvastatin Use and Safety Monitoring in Liver Disease Patients

Simvastatin is mainly metabolized by CYP3A4 [76]. In patients with liver disease, especially those with liver cirrhosis, the liver metabolic function is decreased, and the risk of drug interaction is significantly increased. It is necessary to strictly control the contraindications and precautions of combined use. The core content is shown in the Table 3 below Fig 3.

**Table 3:** Detailed List of Core Drug-Drug Interactions of Simvastatin in Patients with Liver Disease (Recommendations Based on Existing Clinical Studies)

Combined Risk Level	Drug Category/Specific Drugs	Interaction Risk	Clinical Recommended Regimen
Contraindicated combination	Potent CYP3A4 inhibitors: azole antifungals such as ketoconazole and itraconazole; HIV/HCV protease inhibitors; macrolide antibiotics such as erythromycin and clarithromycin; antidepressants such as nefazodone	Significantly increase the blood concentration of simvastatin, greatly increase the risk of myopathy and rhabdomyolysis [87].	It is strictly prohibited to combine. Simvastatin should be discontinued during medication, or statins not metabolized by CYP3A4 should be replaced.
Cautious combination	Rifaximin	There have been cases of self-limited rhabdomyolysis after combined use in patients with decompensated cirrhosis, with potential superimposed risk of myopathy[44, 49]	During the period when the combination is really necessary, closely monitor creatine kinase and muscle symptoms, and strictly limit the dose of simvastatin ≤ 20 mg/d
Cautious combination	Moderate CYP3A4 inhibitors: calcium channel blockers such as amlodipine and diltiazem	Slightly increase the blood concentration of simvastatin and increase the risk of myopathy [91]	The dose of simvastatin should not exceed 20 mg/d during the combination, and strengthen safety monitoring
Safe combination	Carvedilol	The combination has good safety, the incidence of adverse events is no different from that of single drug, and there is no additional adverse effect on systemic hemodynamics [28, 43]	The dose of simvastatin should not exceed 20 mg/d
Safe combination	Metformin	The combination has good safety, can synergistically improve blood lipids and insulin resistance, without superimposed adverse reactions [24]	The dose of simvastatin should not exceed 20 mg/d

### Standardized monitoring scheme for the whole course of medication

#### Comprehensive baseline assessment before medication

Before starting simvastatin treatment, all patients must complete the following examinations to exclude contraindications and clarify the baseline risk:

(1) Complete liver function: ALT/AST, total bilirubin/direct bilirubin, albumin, globulin, INR, prothrombin time, complete Child-Pugh score and MELD score (for patients with liver cirrhosis); (2) Baseline creatine kinase (CK) level; (3) Renal function: serum creatinine, estimated glomerular filtration rate (eGFR), electrolytes; (4) Blood lipid profile: total cholesterol, triglyceride, LDL-C, HDL-C; (5) Comprehensively sort out the combined medication to check the risk of drug interaction; (6) For patients with liver cirrhosis, additional assessment of portal hypertension, ascites, hepatic encephalopathy and other complications is required, and for patients with MASLD, the stage of liver fibrosis and glucose metabolism status need to be assessed.

#### Regular monitoring scheme during medication

**Table:** 

Monitoring Items	Routine Monitoring Frequency	Adjustment for Special Circumstances
Liver function	Recheck 1 month, 3 months, 6 months after medication, and recheck every 6–12 months for long-term medication	For patients with decompensated cirrhosis, shorten to recheck every 1–3 months; recheck must be done within 1 month after dose adjustment
Creatine kinase (CK)	For patients without muscle symptoms, recheck every 6–12 months	Immediate testing when muscle symptoms occur; increase the monitoring frequency when combined with high-risk drugs
Blood lipid and glucose metabolism	Recheck every 3–6 months	For patients with diabetes, synchronously monitor blood glucose and glycosylated hemoglobin
Liver cirrhosis-related complications	Assess every 3–6 months	When the patient has stress states such as infection, trauma, surgery, immediately assess liver function and complications

### Core principles of clinical medication

The hepatoprotective effect of simvastatin cannot replace the basic etiological treatment: patients with MASLD need to adhere to lifestyle intervention, weight management, blood glucose and blood pressure control at the same time [6]; patients with liver cirrhosis need to standardize etiological treatment and complication management, and simvastatin is only used as an adjuvant treatment method [9].

Strictly follow the principle of benefit-risk assessment, only use when the expected benefit is greater than the risk, and strictly prohibit off-indication, off-dose, and off-population medication.

Prioritize the principle of low dose. Patients with decompensated cirrhosis must strictly limit the dose to ≤ 20 mg/d [50], and avoid the risk of serious adverse reactions caused by high-dose use.

Fully perform the obligation of disclosure, and clearly explain to the patient the limitations of existing evidence, especially the risk of off-label drug use and evidence gap in scenarios other than lipid-lowering indications.

## Core limitations of the existing evidence system and future research directions

### Core limitations of the existing evidence system

This section systematically and critically summarizes the evidence shortcomings of simvastatin in the prevention and treatment of the full cycle of chronic liver disease, clarifies the core defects, and points out the direction for future research.

#### Lack of confirmatory evidence-based evidence and unestablished causal relationship

Most of the existing core clinical data supporting the benefits of simvastatin in delaying the progression of fibrosis in MASLD, preventing decompensation in compensated cirrhosis, and primary prevention of HCC are derived from retrospective cohort studies and real-world observational analysis.

Confounding factors and indication bias: Although a number of large-sample studies have adopted statistical methods such as propensity score matching (PSM) [15], they still cannot completely eliminate the interference of unmeasured confounding factors (such as the patient’s baseline BMI, compliance with diet and lifestyle intervention, standardization of basic liver disease management, etc.). In addition, statin users are often accompanied by more frequent medical monitoring, and this inherent indication bias makes the existing data only confirm “correlation”, but cannot establish a clear “causal relationship” between simvastatin and the improvement of long-term clinical hard endpoints.

Doubtful clinical transformation of surrogate endpoints: In some small-sample prospective studies suggesting benefits, most of them use “surrogate endpoints” such as hemodynamics (HVPG), blood lipids, transaminases or insulin resistance index [18, 38]. Whether the improvement of these short-term biochemical or physical indicators can be truly transformed into the improvement of long-term survival rate or the reduction of decompensation/carcinogenesis risk of patients has not been confirmed by high-quality studies.

#### Heterogeneity of core RCT results and limited population extrapolation

The population representativeness of current prospective clinical data has obvious gaps, and blind expansion of the scope of medication or dose has severe clinical risks:

Heterogeneity and red line of benefits in the decompensated stage: In the field of decompensated cirrhosis, the most representative international multicenter phase 3 confirmatory clinical trial (LIVERHOPE study) drew a clear negative conclusion, confirming that simvastatin cannot reduce the risk of ACLF and death in the general population [41]. However, some positive studies suggesting survival benefits (such as the BLEPS study) have their benefit population strictly limited to the narrow population of Child-Pugh grade A/B with a history of variceal bleeding [42, 43]. The significant heterogeneity of results between the two suggests that local benefits cannot be extrapolated to the full compensated/decompensated population.

Data gaps in specific populations: In the field of MASLD, the existing data are highly concentrated in Asian high-risk populations with T2DM or hyperlipidemia [15], and there is an extreme lack of data for patients with non-obese simple steatosis without metabolic comorbidities; in the field of HCC, the secondary prevention of recurrence after radical surgery only has a very small sample of retrospective subgroup tips [60], and the human clinical trial data of combined systemic therapy for advanced stage is completely blank.

Pharmacokinetic black box in high-risk populations: In end-stage patients with severe decompensated stage (Child-Pugh grade C) and MELD score > 12 points, the efficacy of simvastatin is unknown, and it has been confirmed that the toxicity risk of rhabdomyolysis induced by high-dose (≥ 40 mg/d) use will increase sharply. At present, there is a serious lack of pharmacokinetic data in such end-stage populations to guide individualized and precise dose adjustment [50, 53, 54].

#### Transformation gap between preclinical mechanisms and the real human microenvironment

Heavy reliance on preclinical models: Whether it is the anti-fibrosis mechanisms such as reversing hepatic sinusoidal capillarization and inducing ferroptosis of hepatic stellate cells [24, 47, 48], or the anti-HCC effects such as regulating tumor metabolism and reversing targeted drug resistance [61–63], most of the conclusions are derived from rodent animal models and in vitro cell experiments. At present, there is a serious lack of direct verification data on the dynamic changes of real human liver histology, intrahepatic immune microenvironment and tumor microenvironment after simvastatin intervention.

Underestimated complexity of network regulation: Most of the existing mechanism studies focus on the linear verification of single pathways or specific cell subtypes, which may ignore the cross-talk of multiple pathways and compensatory feedback in the complex disease network of the human body. Some pharmacological effects (such as anti-inflammation and immune regulation) have significant model dependence [20, 25], and it is still highly uncertain whether they can be reproduced in human liver diseases driven by different etiologies.

Industrialization barriers of targeted drug delivery systems: Although the novel nano-delivery systems aiming to overcome poor liver/muscle targeting have shown great potential of “enhancing efficacy and reducing toxicity” in animal models [51, 52, 65], there are no related products entering human clinical trials worldwide. The core industrialization bottlenecks such as large-scale production (CMC), long-term in vivo biocompatibility and real targeted enrichment efficiency are still a huge gap before clinical translation.

### Future core research directions and priorities

#### Clinical research priorities

**First Priority: Carry out high-quality prospective RCTs to fill the gap of the highest level of evidence**.

MASLD: Carry out RCTs with liver histological improvement (NAS score, fibrosis stage) and hard endpoints such as decompensation, HCC, and all-cause death as the core; Liver cirrhosis: Evaluate the benefits and risks of long-term decompensation prevention and mortality reduction in patients with compensated and mild to moderate decompensated stage (Child-Pugh A/B); HCC: Give priority to carry out primary prevention RCTs in high-risk populations to confirm its efficacy in preventing new-onset HCC.

**Second Priority: Explore and verify biomarkers for populations with precise benefits**.

Verify the predictive value of markers such as HMGCR expression, cholesterol metabolism, inflammation and fibrosis on the efficacy. Clarify the difference in benefits of populations with different etiologies, disease stages and metabolic characteristics, accurately screen the dominant population, and avoid ineffective medication.

**Third Priority: Verify the effectiveness and safety of combined treatment regimens**.

Based on preclinical synergistic mechanism evidence, carry out standardized clinical trials: In the field of MASLD, explore the combined regimen with hypoglycemic drugs, anti-fibrotic drugs and lifestyle intervention; In the field of liver cirrhosis, verify the combined benefits with non-selective β-blockers, diuretics and etiological treatment drugs; In the field of HCC, confirm the synergistic effect of primary prevention in high-risk populations; for postoperative secondary prevention and advanced combined treatment, early dose and safety exploration should be carried out first.

**Fourth Priority: Focus on special populations and specific disease stages**.

In view of the existing evidence gaps, carry out targeted research: fill the data gaps of special populations such as non-diabetic/non-obese MASLD, women, the elderly and MASLD-related cirrhosis; carry out prospective studies on primary prevention of high-risk populations with acute-on-chronic liver failure (ACLF); clarify the long-term benefits and safety of low-dose medication in patients with decompensated cirrhosis.

**Supplementary Priority: Study on pharmacokinetic characteristics of patients with liver cirrhosis**.

Carry out pharmacokinetic studies of simvastatin in patients with different liver cirrhosis stages and different Child-Pugh grades, clarify the characteristics of drug metabolism, establish an individualized dose adjustment scheme based on liver reserve function, and solve the core problem that the existing dose recommendations lack pharmacokinetic data support.

#### Deepening direction of mechanism research

In-depth analysis of the specific regulatory effects and intercellular interaction mechanisms of simvastatin on different cell subsets in the liver, clarify the basis of differential regulation of diseased cells and normal cells, and provide theoretical support for targeted efficacy enhancement.

Explore the core role and regulatory network of non-apoptotic cell death (ferroptosis, pyroptosis, necroptosis) in its anti-fibrosis and anti-tumor effects, and improve the complete mechanism chain of pharmacological effects.

Systematically analyze the interactive network of core pathways, break through the limitations of single-pathway research, and clarify the correlation and independent mechanisms between the classic lipid-lowering effect and the pleiotropic effects of liver protection and anti-tumor.

Based on the clinical intervention cohort, verify the authenticity of the core preclinical mechanisms in the human body through paired human liver tissue and tumor tissue specimens, and bridge the transformation gap between preclinical and clinical research.

#### Translational research of drug delivery systems

Optimize the validated liver-targeted and tumor-targeted simvastatin nano-delivery systems, complete GLP-level safety evaluation and CMC research, promote the development of phase I clinical trials, and fill the gap in clinical translation.

Develop intelligent drug delivery systems responsive to liver inflammation, fibrosis and tumor microenvironment, realize fixed-point and controllable drug release, further improve targeting, and expand the therapeutic window.

Overcome the core industrialization problems such as large-scale production, quality control and long-term stability of targeted preparations, verify the long-term biocompatibility of the preparations, and lay a foundation for clinical promotion (Table 4).

**Table 4:** Core Evidence Gaps and Future Priority Research Directions of Simvastatin in Chronic Liver Disease

Disease Stage/Research Field	Current Core Evidence Gaps and Clinical Pain Points	First Priority Clinical Research Recommendations (Clinical)	Key Points of Basic and Translational Research (Translational)
Metabolic Dysfunction-Associated Steatotic Liver Disease (MASLD)	Pain points: lack of RCT data on liver histology and long-term hard endpoints; existing evidence is concentrated in diabetic Asian populations, and data for non-obese/non-metabolic comorbidity populations are seriously scarce.	Core actions: Carry out large-sample RCTs with histological improvement (NAS score, fibrosis reversal) and prevention of decompensation as hard endpoints; explore its combined treatment regimen with weight loss and metabolic drugs (such as GLP-1RA).	Translational focus: In human liver specimens, verify the real effect of simvastatin on regulating the polarization of macrophages/CD8 + T cells and lipid metabolism targets (such as DHCR7).
Compensated and Decompensated Liver Cirrhosis	Pain points: the pharmacokinetic (PK) data of patients with decompensated cirrhosis are completely missing, so it is impossible to accurately dose; the existing RCT survival benefits are mostly limited to the narrow population after variceal bleeding, with significant result heterogeneity.	Core actions: Establish an individualized PK model for patients with different liver function reserves (Child-Pugh/MELD grade) to guide precise dose adjustment; carry out large-scale confirmatory RCTs for the prevention of ACLF and reduction of long-term mortality.	Translational focus: Use multi-omics technology to deeply analyze the specific molecular network of targeted regulation of HSC metabolic reprogramming and induction of ferroptosis, and clarify the difference in efficacy under different etiologies.
Hepatocellular Carcinoma (HCC)	Pain points: the whole line (primary prevention, postoperative recurrence prevention, advanced combination) lacks high-quality prospective confirmatory evidence; the conclusions of reversing drug resistance and immune synergy completely stay at the stage of animal and cell models.	Core actions: Give priority to prospective RCTs of primary prevention for high-risk groups of HCC (such as viral hepatitis complicated with hyperlipidemia); promote early dose escalation and safety assessment trials of combined targeted/immunotherapy for advanced HCC.	Translational focus: Clinically verify the reliability of high HMGCR expression, low LDLR expression or specific gene mutations (such as p53 R175H) as predictive biomarkers for precise medication of simvastatin.
Mechanism Transformation and Targeted Drug Delivery System	Pain points: existing mechanisms are heavily dependent on preclinical models, ignoring the compensation of complex human networks; no targeted nano-preparation has entered the clinical stage worldwide, with high industrialization (CMC) barriers.	Core actions: Promote the preclinically validated liver sinusoidal endothelial (LSEC) targeted nano-preparations to complete GLP-level safety evaluation, declare and carry out phase I human clinical trials as soon as possible.	Translational focus: Overcome the core engineering bottlenecks such as large-scale production process, long-term in vivo biocompatibility and real targeted enrichment efficiency of nano drug delivery systems.

## Summary

### Summary of full-text core evidence and mechanisms

Simvastatin is a classic competitive inhibitor of HMG-CoA reductase approved by the FDA, and its overall safety and tolerability have been fully verified after decades of clinical application. In addition to the core lipid-lowering pharmacological effect, the lipid-lowering pleiotropic effects of simvastatin can completely cover the core pathological links of the continuous spectrum of chronic liver disease, providing a potential direction to fill the unmet clinical needs of the full cycle of chronic liver disease (Table 5).

**Table 5:** Map of Core Targeted Pathways of the Hepatic Pleiotropic Effects of Simvastatin

Main Disease Scenarios	Core Targeted Cells	Key Regulatory Pathways/Molecular Targets	Core Biological Effects Produced	Evidence Level
MASLD / Early Liver Injury	Liver sinusoidal endothelial cells (LSEC)	Rac1-Rab7-autophagy-KLF2 positive feedback regulatory loop	Activate autophagic flux, reverse the dedifferentiated phenotype of LSEC capillarization, and improve early microcirculation disturbance.	In vitro cells, animal models
	Hepatocytes	PPARα/PGC-1α signaling pathway; Glucagon receptor (GCGR)	Significantly enhance fatty acid β-oxidation of mitochondria and peroxisomes; reduce cholesterol to reverse glucagon resistance.	In vitro cells, animal models
MASH / Steatohepatitis	Hepatic macrophages	DHCR7-PI3K signaling axis	Reduce intracellular cholesterol levels, restore DHCR7 expression, and reverse pro-inflammatory M1 polarization driven by cholesterol overload.	In vitro, animal, human liver tissue verification
	CD8 + T cells	Intracellular cholesterol synthesis pathway	Reduce the activation of CD8 + T cells, intrahepatic infiltration and secretion of pro-inflammatory factors such as IFN-γ and TNF-α.	In vitro cells, animal models
Liver Fibrosis / Liver Cirrhosis	Liver sinusoidal endothelial cells (LSEC)	KLF2-eNOS signaling pathway	Improve the bioavailability of NO, repair the fenestral structure of hepatic sinuses, and selectively reduce the resistance of intrahepatic microcirculation vessels and portal pressure.	In vitro cells, animal models
	Hepatic stellate cells (HSC)	PKM2/STAT3/c-MYC metabolic axis; GPX4 (ferroptosis pathway)	Inhibit aerobic glycolysis and glutaminolysis of HSCs; target-induced ferroptosis in activated HSCs to block fibrosis.	In vitro, animal, human liver tissue verification
HCC / Hepatocellular Carcinoma	HCC cells (proliferation and apoptosis)	AMPK-p21 and STAT3/Skp2-p27 axis; TAZ (Hippo); ASPP2/SREBP-2	Induce G0/G1 phase arrest of tumor cells; the pro-apoptotic effect is significant in HCC cells with high TAZ expression.	In vitro, in vivo xenograft tumor, human HCC tissue verification
	HCC cells (targeted drug resistance)	HIF-1α/PPAR-γ/PKM2 axis; CASP3-SREBP2-SHH axis	Inhibit the Warburg effect; block cholesterol-driven stemness maintenance, and reverse acquired resistance to sorafenib/lenvatinib.	In vitro, animal, patient-derived organoids (PDO)

In the field of metabolic dysfunction-associated steatotic liver disease (MASLD), large-sample retrospective cohort studies have confirmed that simvastatin can reduce the risk of decompensated liver cirrhosis by 32% and the risk of HCC by 40% in patients with MASLD complicated with T2DM [15], and the protective effect is dose-dependent; evidence from the gold standard of liver biopsy suggests that it can reduce the risk of advanced liver fibrosis by 39% [16]; small-sample clinical studies have shown that it can improve the blood lipid profile and insulin resistance of patients [18]. The core mechanisms include regulating the homeostasis of hepatic lipid metabolism [20, 30], multi-target immune regulation and anti-inflammation [28, 31, 33], inhibiting HSC activation and liver fibrosis progression [8, 21, 24], and improving systemic insulin and glucagon resistance [30, 78], which can block the core pathological chain of MASLD from simple steatosis to steatohepatitis and liver fibrosis.

In the field of liver cirrhosis, evidence-based evidence shows that simvastatin has the clearest benefits in the population with compensated cirrhosis, which can significantly reduce the risk of disease decompensation, HCC and all-cause mortality [35, 36], and at the same time improve liver sinusoidal endothelial function and reduce portal hypertension [38, 48], and prevent variceal bleeding [37]; in patients with mild to moderate decompensated stage (Child-Pugh grade A/B), it can bring significant survival benefits [42, 43], and reduce the risk of ascites and spontaneous bacterial peritonitis [43]; however, the international multicenter phase 3 LIVERHOPE study drew a clear negative conclusion, which did not confirm the disease-modifying benefit of simvastatin in the population with decompensated cirrhosis, and the existing evidence does not support the routine clinical application in this population. Its core mechanisms include targeted regulation of HSC metabolic reprogramming [46], induction of ferroptosis in activated HSCs [47], multi-pathway inhibition of HSC activation to block the progression of liver fibrosis; improvement of LSEC dysfunction through the KLF2-eNOS pathway [24, 48], selective reduction of intrahepatic vascular resistance to alleviate portal hypertension [38, 39]; at the same time, it can inhibit liver and systemic inflammatory response [39, 49], and exert multi-organ protective effects; the nano-targeted delivery system can further improve its liver targeting and expand the therapeutic window [51, 52], but it is still in the preclinical research stage at present.

In the field of HCC, Meta-analysis and large-sample cohort studies have shown that simvastatin exposure is significantly associated with a reduced risk of HCC incidence [56], but the existing clinical evidence is limited to the observational correlation of primary prevention; the secondary prevention of recurrence after radical surgery only has potential benefit tips from subgroup analysis of small-sample retrospective studies [10, 61, 65], and the relevant conclusions of combined therapy for advanced HCC are all from preclinical studies [10, 61, 65], without high-quality human clinical evidence support. Its core anti-tumor mechanisms include inhibiting the malignant transformation of hepatocytes [62, 69], inducing tumor cell cycle arrest and apoptosis [62, 69], correcting the reprogramming of cholesterol metabolism in HCC cells [62, 69], inhibiting the stemness maintenance of liver cancer stem cells [70], reversing targeted therapy resistance [65, 71, 72], remodeling the tumor immunosuppressive microenvironment and inhibiting tumor invasion and metastasis [65, 71, 72].

In terms of safety, the safety of simvastatin in the population with compensated chronic liver disease is similar to that of the general population [50, 54]; in the population with decompensated cirrhosis, adverse reactions are significantly dose-dependent, the dose of 20 mg/d has controllable safety [41], and the dose of 40 mg/d and above will significantly increase the risk of muscle toxicity [41, 54], and the safety risk increases sharply in patients with end-stage liver disease of Child-Pugh grade C and MELD score > 12 points [50, 54]. On the whole, the existing evidence system still has core limitations. The highest level of prospective, multicenter RCT evidence is generally lacking. Most of the benefits of clinical hard endpoints come from observational studies, which can only confirm the correlation but cannot establish a causal relationship. There are significant gaps in evidence for special populations and disease stages [45, 50, 54].

### Concluding remarks

As a classic drug, simvastatin is safe, cheap and easily available. Its “pleiotropic effects” can intervene in the core pathological links of chronic liver disease, and it is a low-cost treatment option with great potential. However, the current efficacy evidence mostly comes from retrospective observations, lacking the support of the highest level of evidence from large-sample, prospective RCTs (randomized controlled trials), and cannot establish an absolute causal relationship; and the evidence is insufficient in the end stage of the disease or special populations. Therefore, blind off-indication medication is strictly prohibited in clinical practice. It must be based on individualized “benefit-risk” assessment, and fully inform patients of the limitations of existing evidence.

In the future, it is urgent to give priority to carrying out high-quality RCT studies with clinical hard endpoints such as liver cirrhosis decompensation, all-cause mortality, and HCC occurrence as the core, to fill the gap of confirmatory evidence; simultaneously deepen the mechanism verification of human tissues, explore precise biomarkers that can predict treatment response, and clarify the optimal benefit population; promote the clinical translation of liver-targeted delivery systems, break through the limitation of the therapeutic window of traditional oral preparations; and finally improve the standardized medication regimen of simvastatin in the whole-cycle management of chronic liver disease, to provide a safer and more effective treatment option for the majority of patients with chronic liver disease.

## Data Availability

No datasets were generated or analysed during the current study.
